# *Pedicularis* L. Genus: Systematics, Botany, Phytochemistry, Chemotaxonomy, Ethnopharmacology, and Other

**DOI:** 10.3390/plants8090306

**Published:** 2019-08-27

**Authors:** Claudio Frezza, Alessandro Venditti, Chiara Toniolo, Daniela De Vita, Ilaria Serafini, Alessandro Ciccòla, Marco Franceschin, Antonio Ventrone, Lamberto Tomassini, Sebastiano Foddai, Marcella Guiso, Marcello Nicoletti, Armandodoriano Bianco, Mauro Serafini

**Affiliations:** 1 Dipartimento di Biologia Ambientale, Università di Roma “La Sapienza”, Piazzale Aldo Moro 5, 00185 Rome, Italy; 2 Dipartimento di Chimica, Università di Roma “La Sapienza”, Piazzale Aldo Moro 5, 00185 Rome, Italy

**Keywords:** *Pedicularis* L. genus, Orobanchaceae family, phytochemistry, chemotaxonomy, ethnopharmacology

## Abstract

In this review, the relevance of the plant species belonging to the *Pedicularis* L. genus has been considered from different points of view. Particular emphasis was given to phytochemistry and ethnopharmacology, since several classes of natural compounds have been reported within this genus and many of its species are well known to be employed in the traditional medicines of many Asian countries. Some important conclusions on the chemotaxonomic and chemosystematic aspects of the genus have also been provided for the first time. Actually, this work represents the first total comprehensive review on this genus.

## 1. Systematics

*Pedicularis* L. is a genus of hemiparasitic plants, originally included in the Scrophulariaceae family but now belonging to the Orobanchaceae family [1]. The rest of the systematic classification is the following: order Scrophulariales, subclass Asteridae, class Magnoliopsida, division Magnoliophyta, superdivision Spermatophyta, subkingdom Tracheobionta. The genus comprises 568 accepted species, 335 synonymous species, and 450 unresolved species [2].

## 2. Etymology of the Name

The etymology of the genus name is Latin, with the term “pediculus” meaning “louse”, which refers to the fact that, according to an ancient English belief, cattle which grazed on these plants were soon found to be infested with lice [3].

## 3. Botany

**Figure 1:**
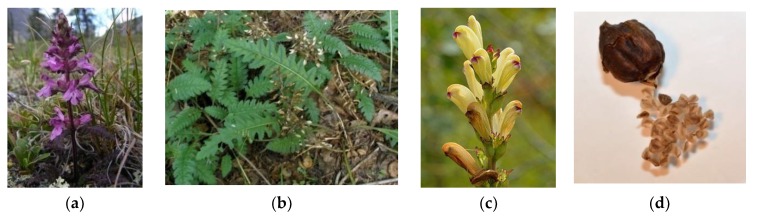
Examples of the morphological features of *Pedicularis* species—stem (**a**), leaves (**b**), flowers (**c**), fruits (**d**) [source Google images].

Plants of the genus *Pedicularis* are generally herbaceous and perennial, with a height which can reach up to 50 cm. Annual or biennial species are quite rare but present. From the morphological standpoint, these species are characterized by big and fleshy roots, often taproots, which contain specific organs (haustoria) for their feeding on the lymph of the near plants. The stem is erect and ascendant and may be simple or branched (Figure 1). The leaves are basal and cauline. The former ones are disposed to form a rosette and are petiolate. The latter ones are opposite, alternated or verticillated, and sessile, instead. Both of these have a lanceolate shape and dentate margins which are rarely entire. Bracts are also present and are similar to the cauline leaves, even if they are smaller (Figure 1). More or less dense terminal spikes generally constitute the inflorescence. The flowers are big, hermaphrodite, zigomorphic, and tetrameric or pentameric. They can be sessile or pedunculated. The floreal formula is X, K(5), [C (2+3), A 2+2], G (2), (superior), capsule. The calyx is gamosepalous, formed by five lobes that may be dentate or not. The corolla is gamopetalous and bilabiate with a cylindrical shape slightly compressed on its sides. Its color ranges from pink to white, passing through red, purple, and yellow. The androecium possesses four didinamous stamens with the filaments well included into the base of the corolla. The anthers are hidden among dense hairs and may be mucronate. The pollen maturation is contemporaneous to the stigma. The ovary is superior, formed by two carpels, and bilocular. The stylus is inserted in the ovary apex and is filiform. The stigma is simple and protruded beyond the corolla hat in order to avoid self-pollination (Figure 1). The fruit is an acuminated bivalve capsule with an oval-lanceolate shape (Figure 1). The seeds are numerous or not and present an angular geometry. Reproduction occurs through pollination by insects or dispersion [4,5].

## 4. Distribution and Habitat

**Figure 2:**
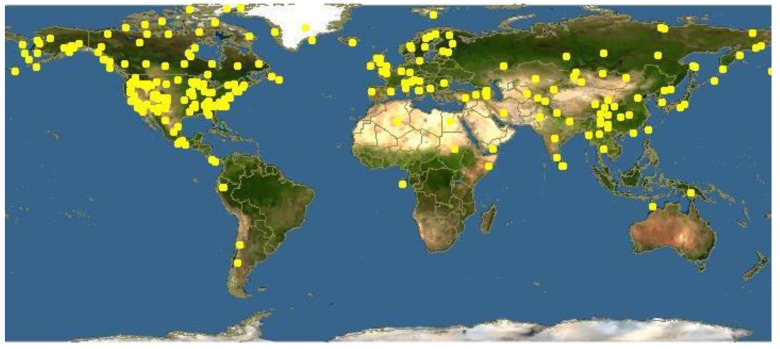
Worldwide distribution of *Pedicularis* species [source Google images].

The species of this genus are distributed in Europe, especially in the mountainous areas of the Mediterranean Basin, and in Northern Asia and America (Figure 2). The highest biodiversity is present in Europe, with about 70 species, India, with about 83 species, and China, with about 350 species, 271 of which are endemic [6,7,8]. In North America, the present species are 36 with two endemisms [9]. These species have been reported in Africa and Australia only as imported plants. The preferred habitat is a temperate mountainous one. The soil must be quite acidic and little- draining. The typical areas where these species can be found are meadows and lawns with little other vegetation [3].

## 5. Phytochemistry

The genus *Pedicularis* is a rich source of different secondary metabolites mainly belonging to the polar fraction. In fact, *Pedicularis* species are poor essential oil producers. Only three species have been investigated as to this aspect, i.e., *Pedicularis condensata* M.Bieb. (u.n.), *P. sibthorpii* Boiss. (a.n.), and *P. wilhelmsiana* Fisch. ex M.Bieb (a.n.). The first one was collected in Turkey and showed the presence of several typical components of essential oils, i.e., more or less oxidized hydrocarbon derivatives and volatile terpenes [10]. The same composition was also observed in the accession of *P. wilhelmsiana* collected in Iran [11]. Indeed, an important difference was found between the two studied exemplars of *P. sibthorpii*, both collected in Iran but in two different regions. In fact, the work by Khodaie et al. [12] did not absolutely evidence the presence of sesquiterpenes, while the work by Morteza-Semnani et al. [13] reported these constituents in high amounts, representing 35.4% of all the identified components. This may actually have been explained by the different environmental growth conditions of the two studied species, which, once again, highlight how essential oil composition is greatly affected by external factors and does not depend only on genetics [14]. 

Among the polar fraction metabolites, several classes of natural compounds were found, i.e., fatty acids, alkaloids, steroids, lignans, *neo*-lignans, tannins, ionones, phenylpropanoid glycosides, phenylethanoid glycosides, flavonoids, xanthones, iridoids, seco-iridoids, phenyl-glycosides, organic acids, polyols, saccharides, and amino acids.

Table 1 reports on the components identified in all studied *Pedicularis* species as reported in literature, according to the species.

As Table 1 clearly shows, only 59 species have been studied for their phytochemical profiles, and, out of these, 12 have been studied only preliminarily, evidencing the presence of some classes of natural compounds but not the specific compounds. 

The highest amounts of identified compounds have been recorded in 14 species, i.e., *P. artselaeri*, *P. chinensis*, *P. decora*, *P. densispica*, *P. dolichocymba*, *P. kansuensis*, *P. longiflora*, *P. longiflora* var. *tubiformis*, *P. muscicola*, *P. rex*, *P. striata*, *P. torta*, *P. tricolor*, and *P. verticillata*, while the lowest amounts have been recorded in 6 species, i.e., *P. acmodonta*, *P. bracteosa*, *P. comosa*, *P. grayi*, *P. sarawchanica*, and *P. semibarbata*. All the other species have been shown to biosynthesize metabolites in medium amounts. In only two cases, the data reported in literature have not specified the organs of the plant species that were studied, i.e., *P. acmodonta* and *P. dolichorrhiza*. 

In general, the studied organs of the plants have been the aerial parts, the leaves, the flowers, or the whole plant, besides a few exceptions, such as *P. chinensis* and *P. grayi*, where the roots have been analyzed, and *P. sarawchanica*, where the fruits have been analyzed. 

Indeed, for what concerns the other accepted, synonymous, and unresolved named species, no phytochemical data or even no data at allare reported in literature. 

Table 2 reports, instead, on the components identified in all the studied *Pedicularis* species, as reported in literature, according to the compound. 

As Table 2 clearly shows, most of the phytochemicals identified in the *Pedicularis* genus belong to the class of natural metabolites known as iridoids. Phenylethanoid glycosides represent the second major class in this context. On the other hand, only one compound belonging to each of the natural classes of alkanes, fatty acids, and coumarins has been isolated from *Pedicularis* spp. 

The iridoid acucubin and the phenylethanoid glycoside verbascoside are the two most common compounds in the entire genus, whereas some cases of specific compounds evidenced in only one species have also been observed. 

As concerns the rest, the presence of other classes of natural metabolites has been shown to be at a medium level, along with their occurrence within the *Pedicularis* genus.

The structures of the majority of the identified compounds in *Pedicularis* species are reported in the figures below (Figure 3, Figure 4, Figure 5, Figure 6, Figure 7, Figure 8, Figure 9, Figure 10, Figure 11, Figure 12, Figure 13, Figure 14, Figure 15, Figure 16, Figure 17, Figure 18, Figure 19, Figure 20 and Figure 21).

## 6. Corollary to Phytochemistry

After visualization of the relative structures of the identified compounds in *Pedicularis* spp., two important elements must be observed and highlighted.

The first one concerns the compound found in *P. kansuensis* by Zhang et al. [50]. According to the structure, the compound should not be named as 1,2,3,16,19,20-hexahydroxyolean-12-en-28-oic acid, but rather as 1,2,3,16,19,20-hexahydroxy-12-ursen-28-oic acid on the basis of the vicinal dimethyl functionalization in positions 19,20 of the pentacyclic triterpene skeleton, which indicates it as an ursane and not an oleane. A similar observation is valid for the 1-(2,3,4-trihydroxyphenyl)ethyl-3-*O*-rhamnose-4-[(2*E*)-3-(3,4-dihydroxyphenyl)-2-propenoate]-glucopyranoside and 1-(2,3,4-trihydroxyphenyl)ethyl-3-*O*-rhamnose-4-[(2*E*)-3-(3,4-dihydroxyphenyl)-2-propenoate]-6-[(2*E*)-3-(3,4-dihydroxy-phenyl)-2-propenoate]-glucopyranoside identified in *P. kansuesnsis* [49,50]. These names were given by the authors, but, actually, according to the routinal numeration of this kind of compounds, they should be named as 1-(,3,4,5-trihydroxyphenyl)ethyl-3-*O*-rhamnose-4-[(2*E*)-3-(3,4-dihydroxyphenyl)-2-propenoate]-glucopyranoside and 1-(3,4,5-trihydroxyphenyl)ethyl-3-*O*-rhamnose-4-[(2*E*)-3-(3,4-dihydroxyphenyl)-2-propenoate]-6-[(2*E*)-3-(3,4-dihydroxyphenyl)-2-propenoate]-glucopyranoside, respectively.

Finally, there are some problems with the correct association between the name of the iridoid longifloroside and its structure, since diverse possibilities are given in the literature. Anyway, in this case, longifloroside is considered to be the compound with the name: 5″-*O*-(4′-aucubinyl)-5′′′*O*-(4′-euphrosidyl)-(2″,2′′′-2,5*H*-furan-ether-(bis-iridoid glucoside), as reported in literature [55].

Moreover, for what concerns iridoids, some of those identified in *Pedicularis* spp. may indeed be artefacts due to the procedures applied during the phytochemical analysis. In particular, the two new iridoid glycosides 6-*O*-ethyl-aucubin and 6-*O*-ethyl-*epi*-aucubin, recognized from *P. rex* [69], and the three pediverticilatasins A–C isolated from *P. verticillata* [87], are likely due to the extraction with ethanol. The same has very likely happened for 6-*O*-methyl-aucubin, artselaenin III, and artselaenin I [19,20], all isolated from *P. artselaeri* after extraction with boiling methanol (at reflux), as well as the 3-butoxy-3,4,dihydroaucubin, 6-*O*-butyl-aucubin, and 6-*O*-butyl-*epi*-aucubin obtained from the *n*-butanol soluble fraction of *P. chinensis* [27]. In this context, the ethyl acetal function observable in the pediverticilatasins A–C and the butyl acetal function of kansuenin B observed in *P. verticillata* [87] are also suspect, in particular if considering that the majority of these compounds have the alcoxy function of the acetal group in α-configuration, which is the opposite of that generally observable for the saccharidic moieties in the glycosidic iridoids. Therefore, the presence of alcoxy acetals could possibly be due to an exchange between the saccharidic moiety and the alcohols present in solution as solvents (thus in high amount), favored by some specific conditions (i.e., acidity of the medium). 

The possibility of generating this kind of artefacts from iridoids was one of the arguments of a recent review and of one editorial article [89,90] which reported about the reactivity of the hydroxyl substituent in allylic configuration, a functionalization very often present in several iridoid structures, like in the case of iridoids with an aucubin-like skeleton, as well as the possibility of addition of short-chain alcohols used as extractive solvents to the double bond in the 3,4-positions of the iridane skeleton. Unfortunately, the presence of such iridoid derivatives was not confirmed in the studied species by avoiding the possible causes of artefact formation. Therefore, the presence of these compounds remains doubtful without any further confirmation.

## 7. Methodologies for the Phytochemical Analysis

The phytochemical analyses of the studied plants were carried out by following the common procedures. In particular, the essential oils were studied through hydrodistillation and gas chromatography (GC) and gas chromatography-mass spectrometry (GC-MS) analysis [10,11,12,13]. 

For the study of the polar fraction metabolites, the starting plant material was mainly dried. The extraction was mainly at room temperature with ethanol, even if extractions with different solvents such as methanol, *n*-hexane, and dichloromethane were also performed. In some cases, these latter extractions were carried out in hot conditions using a Soxhlet apparatus. This extraction method is not the best choice, since the exposure of the extracts to high temperatures may be one cause of artefact formation, as reported in the previous section. Indeed, extraction with ethanol was often followed by a partitioning procedure with solvents at different polarity grades, such as ethyl acetate, n-butanol, diethyl ether, petroleum ether, and distilled water, and every different organic phase was separately subjected to further analysis. The separation of the metabolites from the phytocomplex was mainly achieved by means of column chromatography (CC), using silica gel and allumina as stationary phases and different mixtures of *n*-butanol and distilled water, chloroform or dichloromethane and methanol, or *n*-hexane and ethyl acetate at different concentrations as mobile phases. In a few cases, high performance liquid chromatography (HPLC) techniques were used for these purposes, using C18 columns and distilled water and acetonitrile more or less acidified with formic acid as eluting systems. Identification of the metabolites was mainly achieved by means of thin layer chromatography (TLC), infrared (IR), ultraviolet (UV), optical rotation (OR), nuclear magnetic resonance (NMR), and mass spectrometry (MS) techniques. Finally, preliminary analysis of the metabolite contents was performed via the Folin–Cocalteau test for the total phenolic content (TPC), the aluminium chloride colorimetric assay for the total flavonoid content (TFC), Dragendorff’s reagent test for the presence of alkaloids, and the ferric chloride test for the presence of tannins [15,16,17,18,19,20,21,22,23,24,25,26,27,28,29,30,31,32,33,34,35,36,37,38,39,40,41,42,43,44,45,46,47,48,49,50,51,52,53,54,55,56,57,58,59,60,61,62,63,64,65,66,67,68,69,70,71,72,73,74,75,76,77,78,79,80,81,82,83,84,85,86,87,88]. 

Nonetheless, in a few cases, the methodology was partially or totally undescribed in the reported experimental sections.

At this point, it is extremely important to underline two facts. The first is that the phytochemical methods employed for analysis can deeply influence the results. The second is that works performed only by chromatographic evidence and reporting the generic presence of classes of constituents cannot be considered totally reliable. An example of this is the Dragendorff’s reagent test, which also results positive in the cases of α,β-unsaturated carbonyls.

For these reasons, phytochemical methods must be carefully considered.

## 8. Chemotaxonomy

The chemotaxonomy of the *Pedicularis* genus is quite complex, and involves several classes of natural compounds. In particular, its main chemotaxonomic marker is aucubin, and, in fact, it has been recognized in 25 of the studied species (Table 1). From a biogenetic standpoint, aucubin, like the other decarboxylated C-10 iridoids observed in species of the Lamiales order, derives from geranyl pyrophosphate. In particular, these follow the biosynthetic *Route II*, which involves *epi*-iridotrial and 8-*epi*-deoxy-loganic acid among its precursors, and leads to the biosynthesis of iridoids characterized by the α-configuration of the methyl function linked in the 8 position of the iridane skeleton. Its cyclization reaction occurs through a hydride nucleophillic attack on C1, which leads to the 1-*O*-carbonyl atom attack on C3 and then to the cyclic acetale [91]. Considering the biogenesis of iridoids in this genus, the actual presence of loganic acid recognized among the phytoconstituents of *P. torta* and *P. longiflora* is doubtful (see table for references). We are instead of the opinion that, without further confirmation, that compound was mistakenly reported instead of *epi*-loganic acid. In this context, studies on the biogenesis of iridoids in *Pedicularis* spp. by means of labelled precursors could be of help in delineating the biogenetic pathway and several products of that metabolite biosynthesis. This could also be an excellent analytical method by which to confirm the possible presence or not of compounds that appear to be in contrast with the biogenetic pathway of iridoids in this genus.

In fact, euphroside and mussaneoside are also minor chemotaxonomic markers of the genus, even if in several species the content of euphroside was shown to be higher than that of aucubin itself [22,51], and the amount of mussaenosidic acid was comparable with those of other iridoidic constituents [60]. Conversely, some iridoids are considered to be chemotaxonomic markers at the species level, since their presence has been reported only in one. The main example of this are pedicularioside for *P. muscicola*, kansuenin, kansuenin B, and kansuenoside for *P. kansuensis*, pliatosides A–B for *P. plicata*, and densispicnin A for *P. densispica*. In contrast with what is written in the previous paragraph concerning artefact iridoids, the presence of proceroside in *P. procera* [66], even if it presents a β-configuration in C-8 and therefore would seem to be derived from the Route I biogenetic pathway, is not an artefact and is not due to an erroneous interpretation of experimental data. In fact, the inversion of configuration at C-8 in proceroside is favored by the presence of a ketone function on the adjacent carbon (C-7), which is involved in a keto-enol equilibrium, and this may perfectly justify the β-configuration of the hydroxymethyl group at the position 8.

*Seco*-iridoids are metabolites that rarely derive from the biogenetic *Route II*. In fact, some derivatives have been observed in *Lamium album* [92], and their origin from 8-*epi*-deoxy-loganic acid, a precursor in the biogenetic *Route II*, has been fully confirmed. To date, the presence of *seco*-iridoids has been observed only in *P. verticillata* [70], and it could be of utmost interest to verify if these kinds of compounds are also present in other species of the genus. Obviously, it could also be interesting to investigate their possible biogenesis by suitable analytical methods.

Phenylethanoid glycosides (i.e., verbascoside and its derivatives) are considered to be other chemotaxonomic markers of the genus, since their presence has been evidenced in most of the studied species. However, these compounds are very common in all the Asteridae class, and, in fact, they have also been identified in other families such as Asteraceae [93], Caprifoliaceae [94], Lamiaceae [14], Oleaceae [95], Plantaginaceae [96], Scrophulariaceae [97], and Verbenaceae [98]. More specifically, the phenylethanoid glycosides have a chemotaxonomical relevance when co-occurring with iridoids [99]. This has been already observed in several species in the Lamiales order [100,101,102,103,104,105], as well as in the case of several *Pedicularis* spp. These compounds are also extremely common within the family *Pedicularis* genus belongs to (Orobanchaceae), and, in fact, they have already been reported in several genera, such as *Orobanche* L., *Cistanche* L., and *Orthocarpus* Nutt. [106]. For these reasons, phenylethanoid glycosides cannot actually be taken as general chemotaxonomic markers of the *Pedicularis* genus. Nevertheless, specific compounds can be useful chemotaxonomic markers, such as pediculariosides A, E, G, H, I, M, and N for the entire genus, permethyl-verbascoside for *P. spicata*, *cis*-*iso*-martynoside for *P. kansuensis*, *cis*-pedicularioside H for *P. spicata*, and artselaeroside B for *P. artselaeri*. 

Lignans and derivatives are quite widespread in the genus, but also in the family Orobanchaceae and in many others [107]. However, semitortosides A and B can serve as chemotaxonomic markers for *P. semitorta*, striatosides A and B can serve as chemotaxonomic markers for *P. striata*, and longiflor B and longiflorides C and D can serve as chemotaxonomic markers for *P. longiflora*.

Flavones and, in particular, flavonols and glycosidic flavonoids presenting an apigenin, scutellarein, and isoscutellarein base moiety, are also considered to be chemotaxonomic markers of the genus. However, they are very common compounds in the plant kingdom, and for this reason, they are not particularly useful as chemotaxonomic markers. In particular, their presence can be easily evidenced in Lamiaceae species [14], as well as in many other families, such as Euphorbiaceae, Asteraceae, Compositae, and Hypericaceae [108,109,110,111,112,113,114].

In terms of alkaloids, pediculidine, pedicularidine, pediculine, and pediculinine have been evidenced only in *Pedicularis* species, and they can serve as chemotaxonomic markers at the genus level.

As for compounds belonging to classes of natural metabolites other than the ones already described, there have been no reports on them as chemotaxonomic markers of the *Pedicularis* genus or in general, since they are extremely common. Nevertheless, pedicurexoside, a sesquiterpene, may be suggested as a specific marker for *P. rex*, since it has been evidenced only in that species so far, while the polyol D-mannitol seems to be highly represented in hemiparasitic entities previously comprised in Scrophulariaceae and now classified as Orobanchaceae [51,70,102,115]. 

In this context, concerning phytochemistry and chemotaxonomy, it is of primary importance to also consider other aspects, together with the markers’ metabolite biogenesis, such as the ecology and hemiparasitic behaviour of the plant species, when the scope of the study is chemosystematics. In fact, the transfer of metabolites from the hosts to the hemiparasitic species has been observed in several cases, such in the cases of *Euphrasia stricta* D. Wolff [116], *Euphrasia rostkoviana* Hayne [117], and *Odontites luteus* Steven [118]. Therefore, it is suggested that the results from the phytochemical analysis of hemiparasitic plants should be carefully checked and subjected to the required criticism.

## 9. Ethnopharmacology

*Pedicularis* species are widely used in the traditional medicines of several countries around the world, especially Asian ones. The pharmacological activities exerted by these species are numerous and interesting, with one species often employed to treat more than one malady and vice versa. 

Table 3 reports on the specific ethnopharmacological properties associated with every studied plant in this field. In addition, the organs of the plant species which show that medicinal activity are described, as well as the areas of the world where indigenous people employ these species in traditional medicine.

## 10. Corollary for Ethnopharmacology

Some *Pedicularis* species have also been reported to have ethnopharmacological employments in certain areas of the world, but no specific medicinal and pharmacological properties have been reported in the literature. In particular, this concerns *P. koengboensis* Tsoong var. *kongboensis* (a.n.) in Nepal [151], *P. heydei* Prain (u.n.), *P. nodosa* Pennell (u.n.) and *P. scullyana* Prain ex. Maxim. (u.n.) in Tibet [152], and, finally, *P. tristis* L. (a.n.) in Mongolia [153]. Specific information concerning their specific way of employment is also lacking in the literature, which makes their uses doubtful but not certainly false, since their utilization may be only on a traditional local basis and favored by specialized people who may not be interested in sharing their knowledge. Regardless, phytochemical analysis of these species is also strongly suggested in the future.

## 11. Pharmacology

In spite of all the results reported in the previous section, for some *Pedicularis* species, only a few initial pharmacological properties have been assessed, and their ethnopharmacological employments have not yet been reported. This also concerns the species already used in the ethnopharmacological field but that have been studied for other possible employments. 

Table 4 reports on these species and their relative pharmacological properties.

## 12. Relationships among Pharmacology, Ethnopharmacology, and Phytochemistry

Table 2 and Table 3 clearly show how fundamental *Pedicularis* species are in the ethnopharmacological and pharmacological fields. However, many *Pedicularis* species with ethnopharmacological and/or pharmacological uses are awaiting phytochemical analysis on their active constituents. Thus, their employment is strictly related to traditional uses, which are established on the basis of previous experiences. Conversely, for those species also presenting a well-established phytochemical profile, their ethnopharmacological and/or pharmacological uses can be obviously explained by their phytochemical compositions. In fact, phytochemical compounds (singularly or as a phytocomplex) are the major elements responsible for the pharmacological properties associated to every single species, and may justify their use in that sense from the phytochemical standpoint.

Several classes of natural compounds have been evidenced within the *Pedicularis* genus, and each of them exerts specific pharmacological activities. In particular, alkaloids have antimalarial, antitumor, antibacterial, and stimulant activities, among others [154,161], even if a particular subclass of them (pyrrolizidine alkaloids) are indeed known to cause severe genotoxicity, neurotoxicity, and tumourigenicity [162]. Lignans exert mainly antioxidant and anti-inflammatory properties [163]. Tannins are widely known for their astringent and antioxidant effects [164]. Phenylethanoid glycosides are good antioxidant, antibacterial, antiviral, antitumor, neuroprotective, and hepatoprotective compounds [106,165]. Flavonoids display, in particular, antioxidant, anti-inflammatory, anti-mutagenic, and anti-carcinogenic properties [166]. Xanthones are mainly insecticidal compounds [167]. Iridoids are widely used as antiviral, anti-inflammatory, hepatoprotective, antimicrobial, and antitumor agents [168]. *Seco*-iridoids are mainly anti-inflammatory and antifungal compounds [169]. Finally, fatty acids, organic acids, polyols, saccharides, nucleobases, and amino acids have several nutraceutical properties.

## 13. Other Uses

Some *Pedicularis* species are better known to have other uses different from those typical in the ethnopharmacological and pharmacological fields.

These uses all are reported in the table below (Table 5).

## 14. Curiosities

Some *Pedicularis* species present strange but interesting curiosities. In particular, although *Pedicularis* species are considered to be strong hemiparasitic plants, *P. friderici-augusti* Tomm. (a.n.), *P. furbishiae* S. Watson (a.n.), *P. ishidoyana* Koidz. & Ohwi (u.n.), *P. kashmiriana* Pennell (a.n.), *P. petiolaris* Ten. (a.n.), *P. rainierensis* Pennel & Warren (a.n.), *P. rostratospicata* Crantz (a.n.), *P. siamensis* P.C.Tsoong (u.n.), and *P. thailandica* T.Yamaz. (u.n.) are endangered species in their growth areas [173,174,175,176,177,178,179]. Moreover, *P. porrecta* Wall. (u.n.) grows only in arid areas [180], and the name *P. stenantha* Franch. (u.n.) is also often used to identify *P. stenocorys* Franch. (a.n.), but they are two different species [181].

## 15. Conclusions

This review has clearly evidenced and highlighted the importance of the plant species belonging to the *Pedicularis* genus from different points of view.

As it can be easily deduced, there is still much to discover and study, since the information about this genus is quite scarce as regards many specific arguments.

In particular, it could be interesting to investigate the biogenesis of iridoids, since, from a chemosystematic standpoint, they are the most important marker compounds in this genus. This may confirm or not the presence of unusual compounds such as derivatives with 8β-configurations and *seco*-iridoids, as well as potentially elucidate the key intermediates in their biosynthesis by means of labeled precursors.

On the other hand, for what concerns the bioactivity aspects of *Pedicularis* spp., we hope that this review will contribute to renewing the interest of researchers in deepening the general knowledge on the pharmacological potentials of *Pedicularis* extracts and pure constituents, in particular, their minor components.

## Figures and Tables

**Figure 3:**
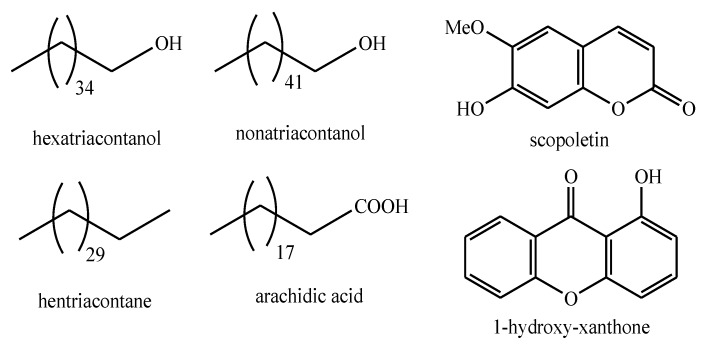
Fatty acids, alkanes, alkyl alcohols, coumarins, and xanthones identified in *Pedicularis* species.

**Figure 4:**
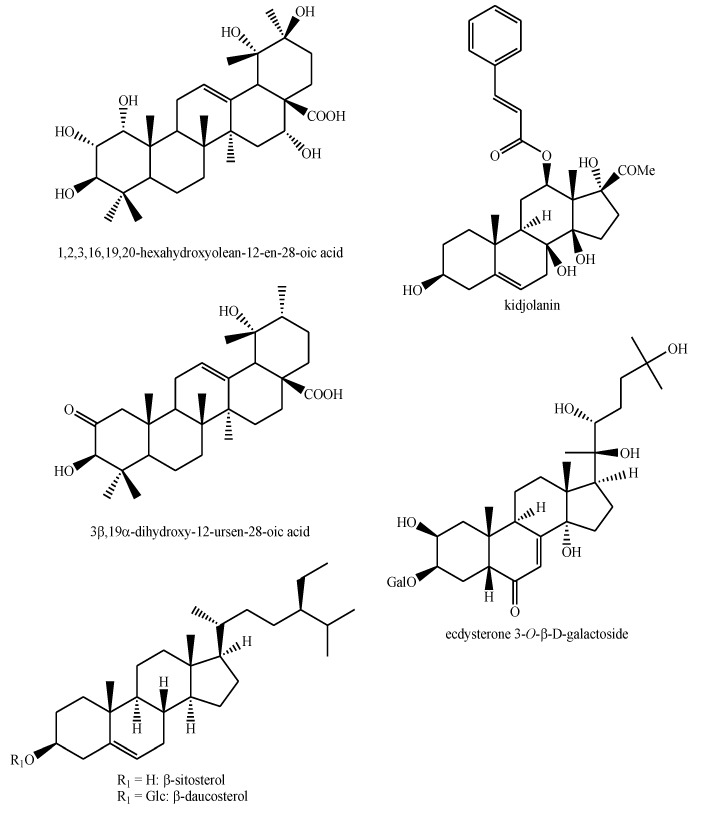
Terpenoids identified in *Pedicularis* species.

**Figure 5:**
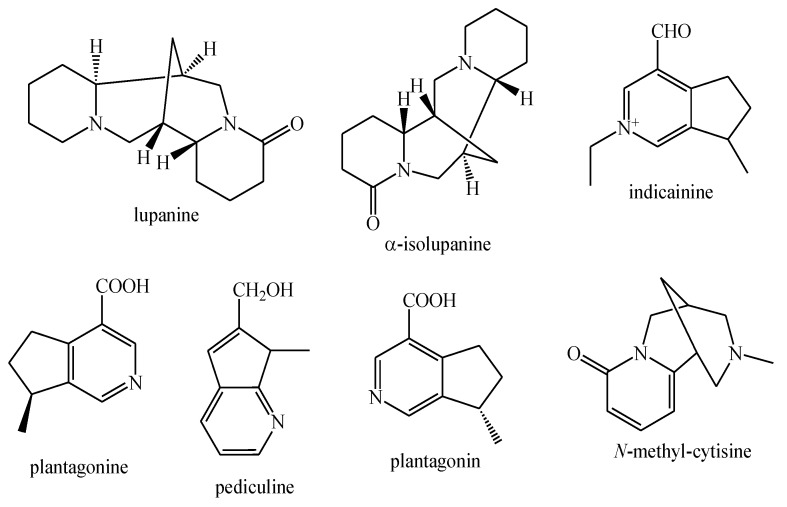
Alkaloids identified in *Pedicularis* species—part 1.

**Figure 6:**
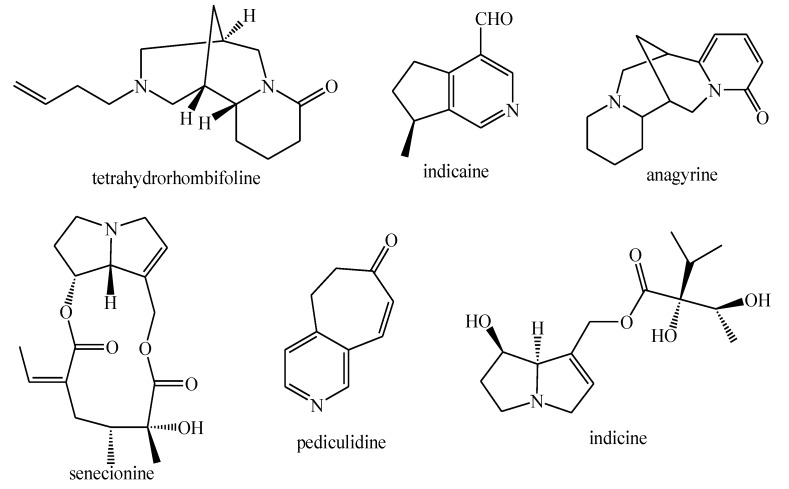
Alkaloids identified in *Pedicularis* species—part 2.

**Figure 7:**
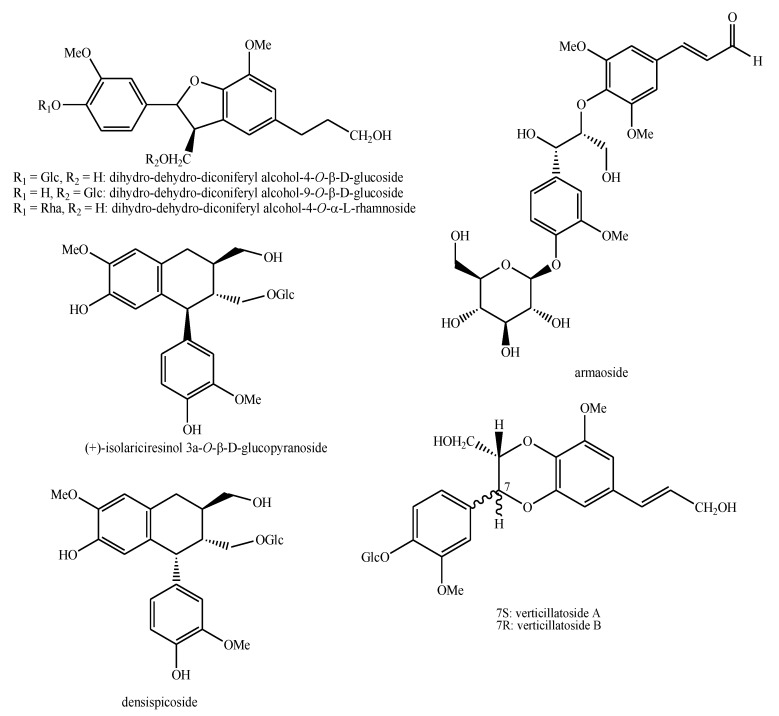
Lignans and *neo*-lignans identified in *Pedicularis* species—part 1.

**Figure 8:**
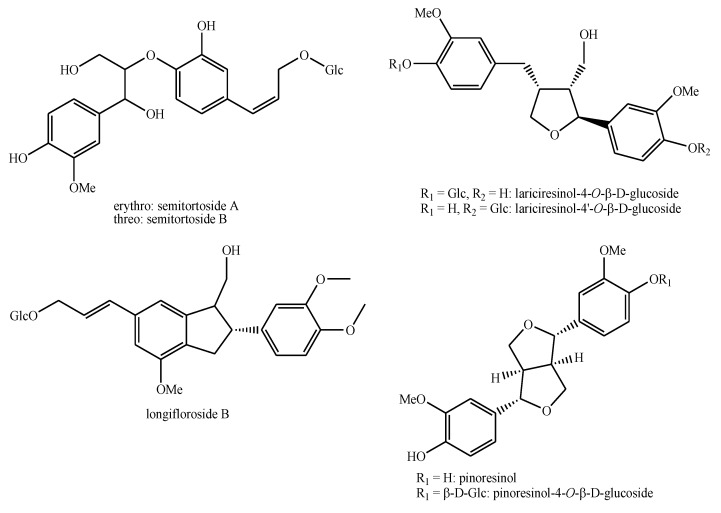
Lignans and *neo*-lignans identified in *Pedicularis* species—part 2.

**Figure 9:**
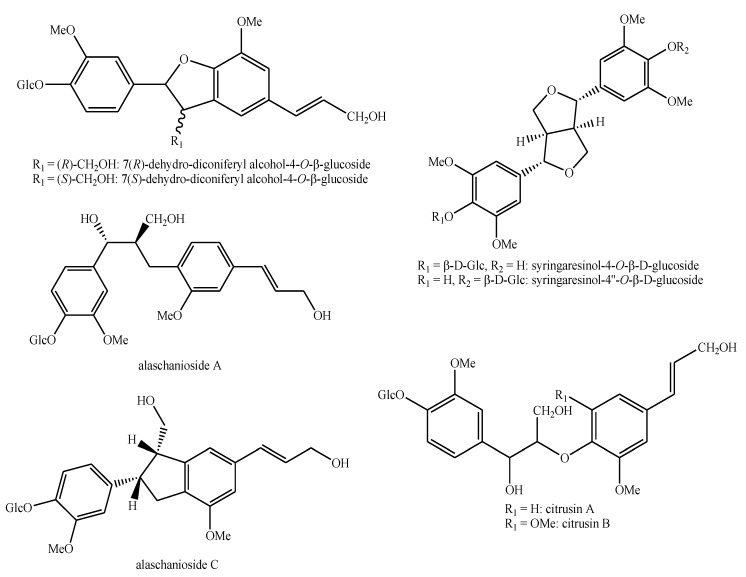
Lignans and *neo*-lignans identified in *Pedicularis* species—part 3.

**Figure 10:**
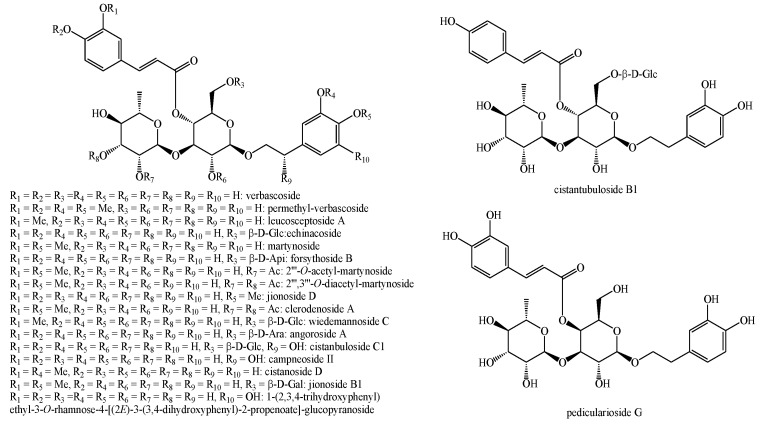
Phenylethanoid glycosides identified in *Pedicularis* species—part 1.

**Figure 11:**
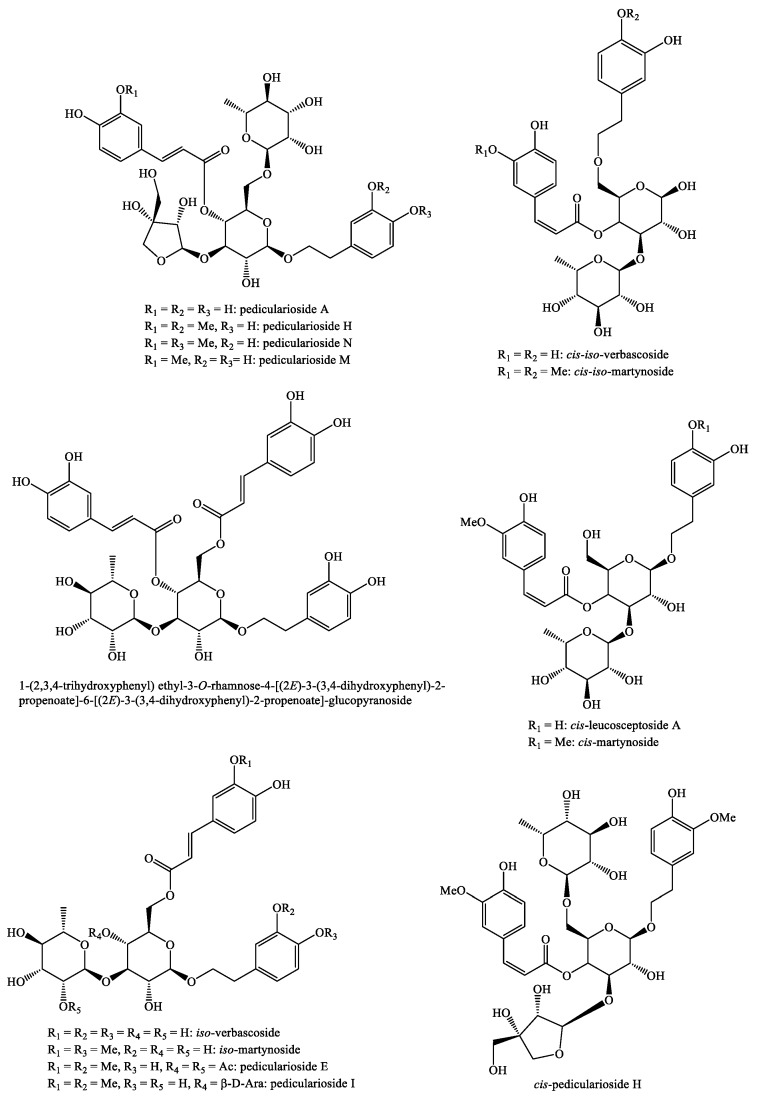
Phenylethanoid glycosides identified in *Pedicularis* species—part 2.

**Figure 12:**
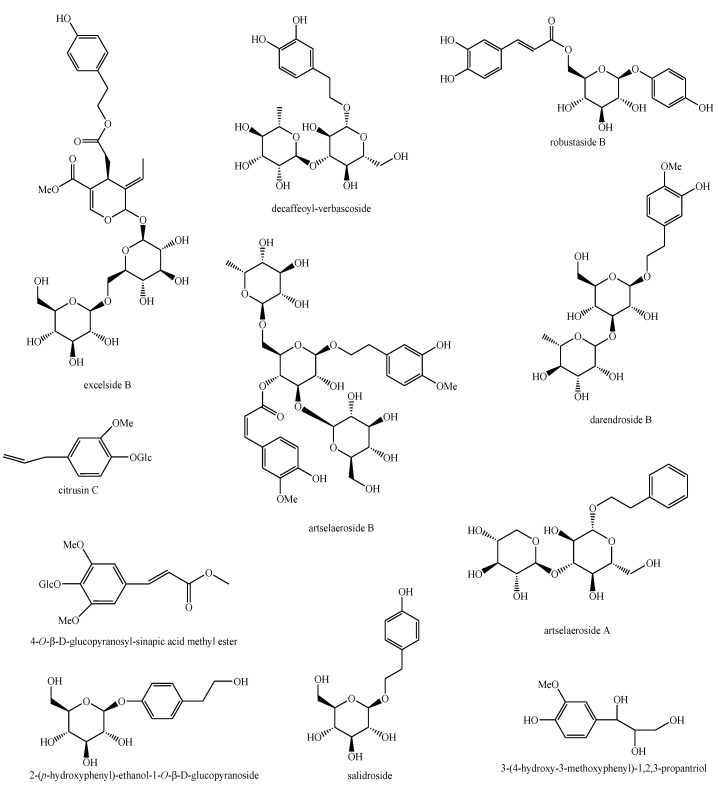
Phenylethanoid glycosides identified in *Pedicularis* species—part 3.

**Figure 13:**
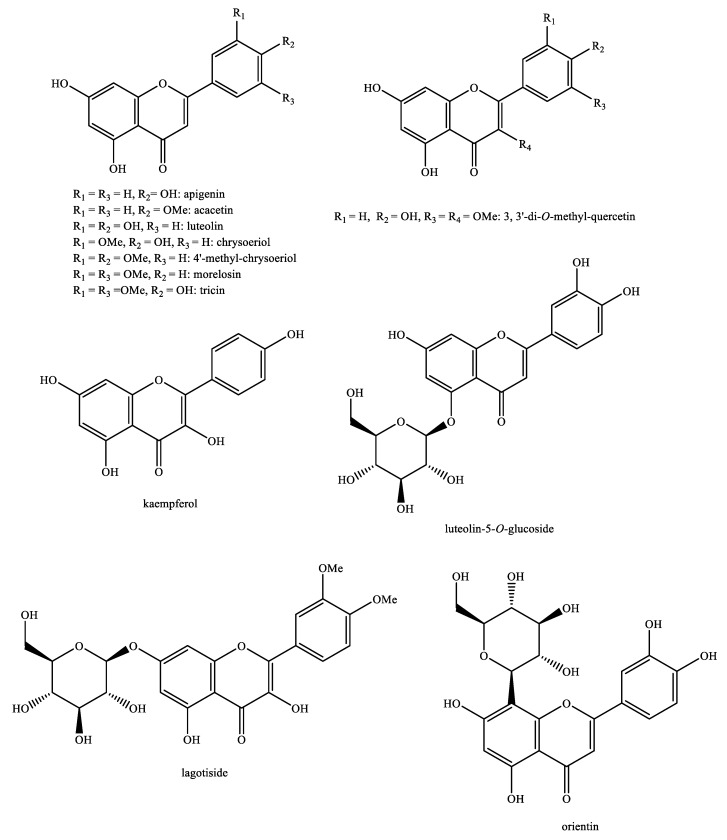
Flavonoids identified in *Pedicularis* species—part 1.

**Figure 14:**
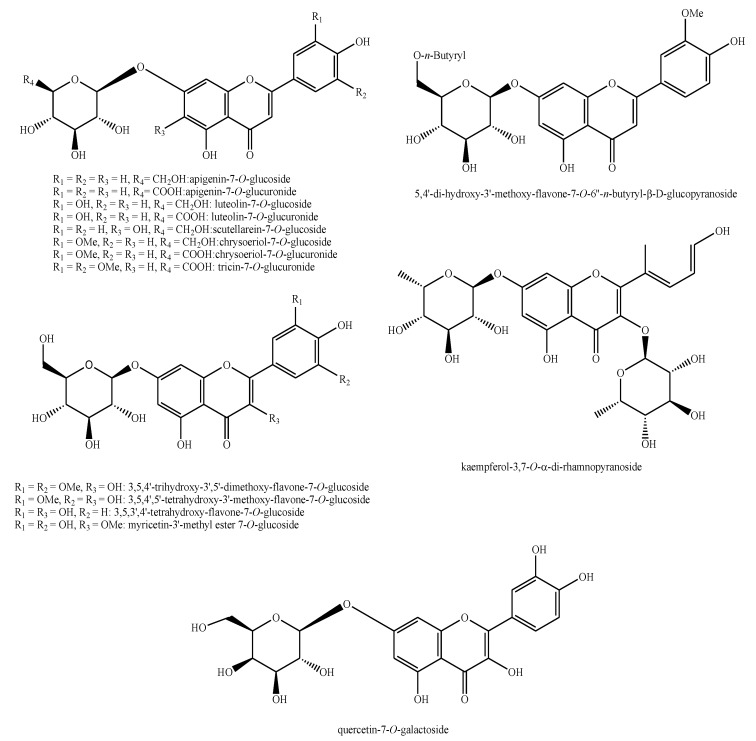
Flavonoids identified in *Pedicularis* species—part 2.

**Figure 15:**
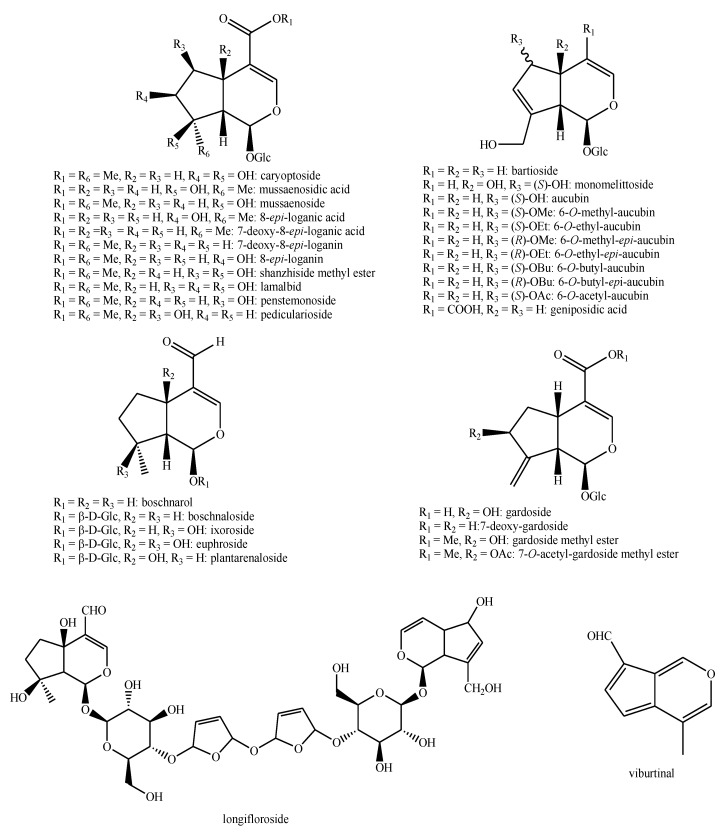
Iridoids identified in *Pedicularis* species—part 1.

**Figure 16:**
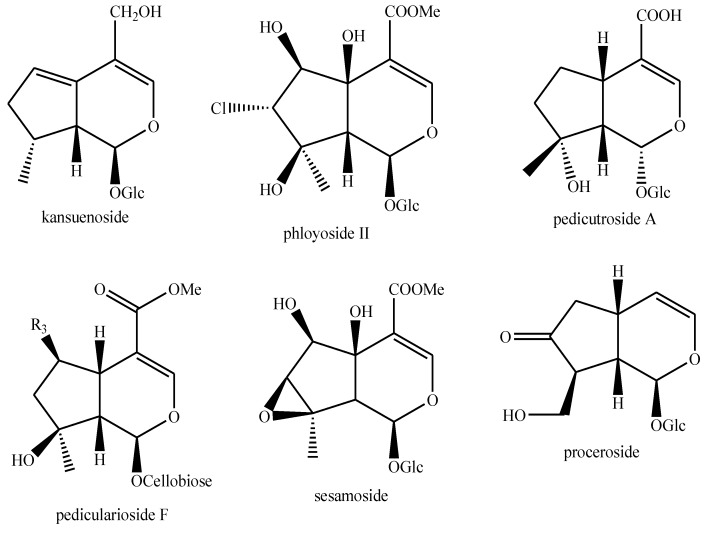
Iridoids identified in *Pedicularis* species—part 2.

**Figure 17:**
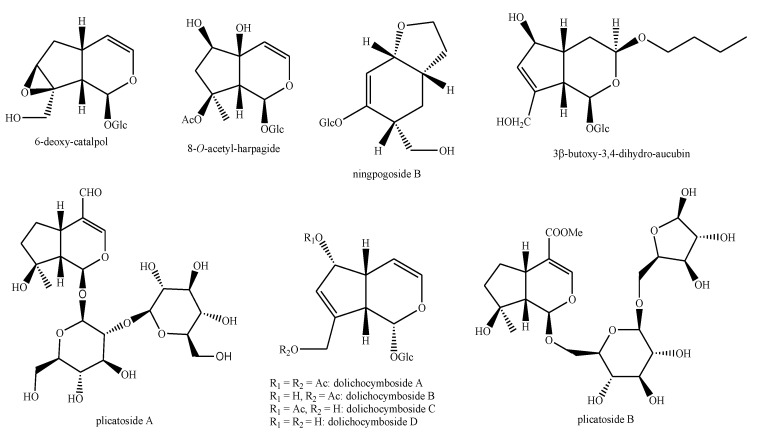
Iridoids identified in *Pedicularis* species—part 3.

**Figure 18:**
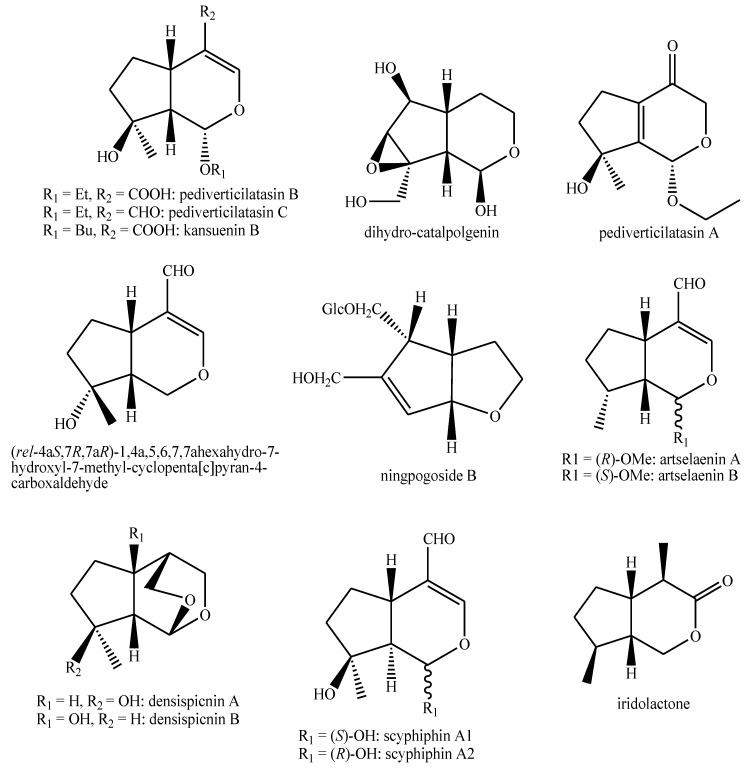
Iridoids identified in *Pedicularis* species—part 4.

**Figure 19:**
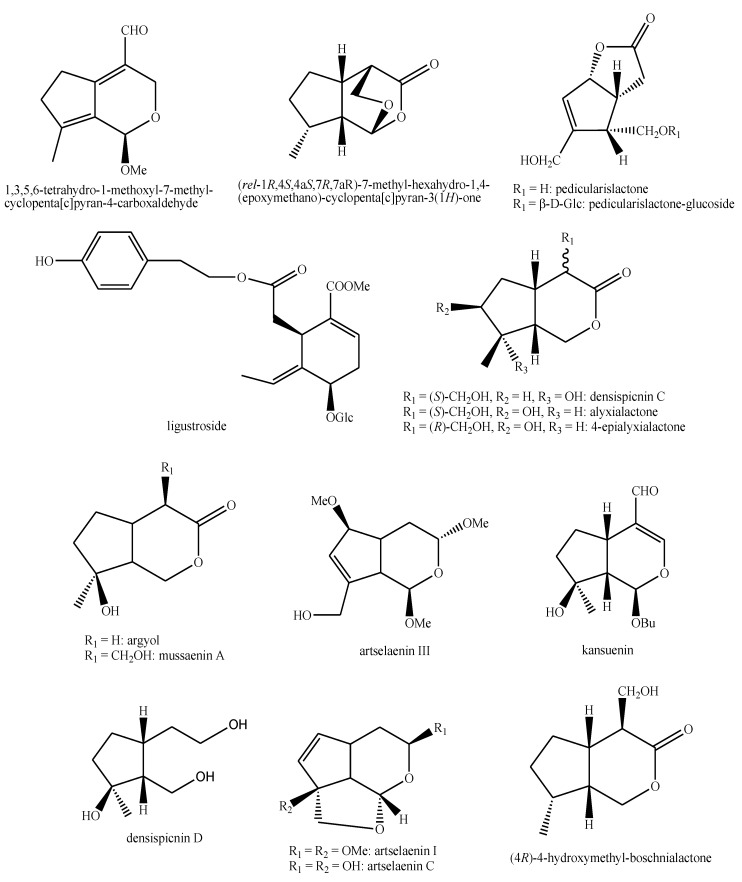
Iridoids identified in *Pedicularis* species—part 5.

**Figure 20:**
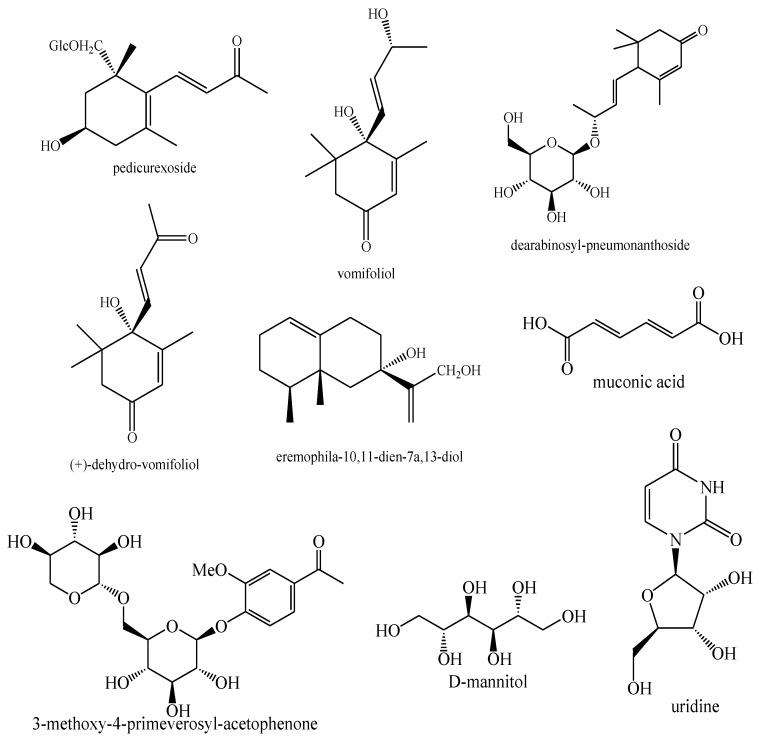
Other compounds identified in *Pedicularis* species—part 1.

**Figure 21:**
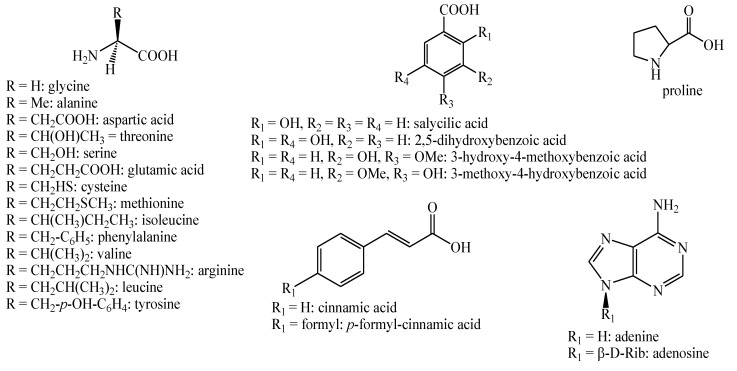
Other compounds identified in *Pedicularis* species—part 2.

**Table 1:** Phytochemical Compounds Reported in the Studied *Pedicularis* Species.

Pedicularis spp.	Studied Organs	Phytochemical Compounds	References
*P. acmodonta* Boiss. (u.n.)	n.s.	leucosceptoside A, echinacoside	[15]
*P. alaschanica* Maxim. (a.n.)	aerial parts	alaschanioside A, alaschanioside C, citrusin A, syringaresinol-4-*O*-β-d-glucoside, verbascoside, leucosceptoside A, martynoside, boschnaloside, ixoroside, euphroside, geniposidic acid, mussaenosidic acid	[16,17]
*P. armata* Maxim (a.n.)	whole plant	armaoside, citrusin B, euphroside, mussaenoside, geniposidic acid, 8-*epi*-loganic acid, aucubin	[18]
*P. artselaeri* Maxim. (a.n.)	whole plant	lariciresinol-4-*O*-β-d-glucoside, lariciresinol-4′-*O*-β-d-glucoside, alaschanioside A, citrusin A, artselaeroside A, artselaeroside B, 2-(*p*-hydroxyphenyl)-ethanol-1-*O*-β-d-glucopyranoside, *iso*-verbascoside, martynoside, artselaenin I, artselaenin III, artselaenin A, artselaenin B, artselaenin C, 8-*epi*-loganic acid, 8-*epi*-loganin, 7-deoxy-8-*epi*-loganic acid, plantarenaloside, mussaenoside, aucubin, 6-*O*-methyl-aucubin, 6-*O*-methyl-*epi*-aucubin, ixoroside, 7-deoxy-gardoside, gardoside methyl ester, caryoptoside, shanzhiside methyl ester, 3-methoxy-4-primeverosyl-acetophenone	[19,20]
*P. bicornuta* Klotzsch (u.n.)	whole plant	alkaloids, lignans glycosides, phenylpropanoid glycosides, flavonoids, iridoids (**exact compounds not specified**)	[21]
*P. bracteosa* Benth. (a.n.)	aerial parts	aucubin, mussaenoside	[22]
*P. bracteosa* subsp. paysoniana (Pennell) W.A. Weber (a.n.)	whole plant	alkaloids (**exact compounds not specified**)	[23]
*P. capitata* Adams (a.n.)	leaves	alkaloids (**exact compounds not specified**)	[24]
*P. cephalantha* Franch. ex. Maxim. (a.n.)	whole plant	kidjolanin, pinoresinol, martynoside, *iso*-martynoside, clerodenoside A, acacetin, luteolin, 7-deoxy-gardoside, plantarenaloside, mussaenosidic acid, euphroside, mussaenoside, aucubin	[25]
*P. chamissonis* Steven (a.n.)	leaves	verbascoside, luteolin-7-*O*-glucoside, luteolin-7-*O*-glucuronide	[26]
*P. chinensis* Maxim. (a.n.)	roots	syringaresinol-4-*O*-β-d-glucoside, martynoside, *cis*-martynoside, pedicularioside N, 1-*O*-β-d-(3-hydroxy-4-methoxy-phenyl)-ethyl-β-d-apiosyl-l-(1→3)-rhamnosyl-(1→6)-4-*trans*-feruloyl-glucopyranoside, 1-*O*-β-d-(3-hydroxy-4-methoxy-phenyl)-ethyl-β-1-(1→3)-4-*trans*-feruloyl-glucopyranoside, 1-*O*-β-d-(3-hydroxy-4-methoxy-phenyl)-ethyl-α-l-rhamnosyl(1→3)-4-*cis*-feruloyl-gulopyranoside, luteolin-7-*O*-glucoside, aucubin, 6-*O*-methyl-aucubin, 6-*O*-butyl-aucubin, 3β-butoxy-3,4-dihydro-aucubin, 6-*O*-butyl-*epi*-aucubin, iridolactone, bartsioside, pedicularislactone, pedicularislactone glucoside, *rel*-(6*R*,5*R*,9*R*)-(2-oxa-bicyclo-[3,3,0]oct-3-one-8-en-9,8-diyl)-dimethanol	[27,28]
*P. comosa* L. (a.n.)	aerial parts	verbascoside, forsythoside B	[15]
*P. condensata*	aerial parts	verbascoside, echinacoside, aucubin, 6-*O*-acetyl-aucubin, 8-*epi*-loganin, mussaenoside, shanzhiside methyl ester, gardoside methyl ester	[29]
*P. crenulata* Benth. (a.n.)	aerial parts	anagyrine, aucubin, euphroside, plantarenaloside	[22,30]
*P. decora* Franch. (a.n.)	whole plant	β-sitosterol, β-daucosterol, *iso*-verbascoside, kaempferol, aucubin, lamalbid, pedicularislactone glucoside, ningpogoside B, d-mannitol, β-(3′,4′-dihydroxyphenyl-*O*-a-l-rhamnopyranosyl- (1–3)-β-d-glucopyranoside, salicylic acid, 2,5-dihydroxybenzoic acid, 3-hydroxy-4-methoxybenzoic acid, 3-methoxy-4-hydroxybenzoic acid, aspartic acid, threonine, serine, glutamic acid, glycine, alanine, cysteine, methionine, isoleucine, phenylalanine, valine, arginine, proline, leucine, tyrosine	[31,32,33,34,35,36]
*P. densispica* Franch. ex. Maxim. (a.n.)	whole plant	pedicutricone A, (+)-isolariciresinol 3a-*O*-β-d-glucopyranoside, pinoresinol-4-*O*-β-d-glucoside, syringaresinol-4-*O*-β-d-glucoside, longifloroside B, densispicoside, verbascoside, martynoside, *iso*-martynoside, 2″-*O*-acetyl-verbascoside, *cis*-martynoside, salidroside, darendoside B, 4-*O*-β-d-glucopyranosyl-sinapic acid methyl ester, 3-(4-hydroxy-3-methoxyphenyl)-1,2,3-propantriol, citrusin C, robustaside B, acacetin, kaempferol, apigenin-7-*O*-glucoside, kaempferol-3,7-*O*-α-di-rhamnopyranoside, scutellarein-7-*O*-glucoside, chrysoeriol-7-*O*-glucoside, mussaenin A, mussaenoside, argyol, densispicnin A, densispicnin B, densispicnin C, densispicnin D, shanzhiside methyl ester, 8-*epi*-loganin, dearabinosyl-pneumonanthoside, maltol-β-d-glucoside	[37,38,39]
*P. dolichocymba* Hand.-Mazz. (a.n.)	whole plant	plantagonine, indicaine, pediculidine, pediculine, lariciresinol-4′-*O*-β-d-glucoside, verbascoside, martynoside, 2′′′-*O*-acetyl-martynoside, leucosceptoside A, jionoside D, 2-phenylethyl-*O*-β-d-xylopyranosyl-(1→2)-β-d-glucopyranoside, benzyl alcohol-*O*-β-d-xylopyranosyl-(1→2)-β-d-glucopyranoside, apigenin, dolichocymboside A, dolichocymboside B, dolichocymboside C, dolichocymboside D, gardoside methyl ester, 7-*O*-acetyl-gardoside methyl ester, uridine, adenosine	[30,40,41,42]
*P. dolichorrhiza* Schrenk (a.n.)	n.s.	plantagonine, indicaine, pediculidine, pediculine	[42]
*P. gracilis* Wall. ex. Benth. (a.n.)	whole plant	tannins, terpenoids, flavonoids, glycosides (**exact compounds not specified**)	[43]
*P. grayi* A. Nelson (a.n.)	roots	*N*-methyl-cytisine	[30]
*P. groenlandica* Retz. (a.n.)	aerial parts	senecionine, aucubin, euphroside, mussaenoside	[22,30]
*P. integrifolia* Hook. f. (a.n.)	aerial parts	alkaloids, tannins (**exact compounds not specified**)	[44]
*P. kanei* Durand (s.n.)	leaves	alkaloids (**exact compounds not specified**)	[24]
*P. kansuensis* Maxim. (a.n.)	whole plant	β-sitosterol, β-daucosterol, 1,2,3,16,19,20-hexahydroxyolean-12-en-28-oic acid, alaschanioside A, alaschanioside C, verbascoside, leucosceptoside A, martynoside, *iso*-martynoside, *cis*-*iso*-martynoside, 2′′′,3′′′-*O*-diacetyl-martynoside, jionoside B1, pedicularioside A, pedicularioside M, echinacoside, forsythoside B, phenethylalcohol β-sophoroside, 1-(2,3,4-trihydroxyphenyl)ethyl-3-*O*-rhamnose-4-[(2*E*)-3-(3,4-dihydroxyphenyl)-2-propenoate]-glucopyranoside, 1-(2,3,4-trihydroxyphenyl)ethyl-3-*O*-rhamnose-4-[(2*E*)-3-(3,4-dihydroxyphenyl)-2-propenoate]-6-[(2*E*)-3-(3,4-dihydroxyphenyl)-2-propenoate]-glucopyranoside, 4′-methyl-chrysoeriol, luteolin, luteolin-7-*O*-glucoside, lagotiside, tricin-7-*O*-glucuronide, kansuenin, kansuenin B, kansuenoside, ixoroside, gardoside methyl ester, geniposidic acid, euphroside, mussaenoside, boschnaloside, 7-deoxy-8-*epi*-loganic acid, 8-*epi*-loganic acid, aucubin, geniposidic acid, (*E*)-2-hexenyl β-sophoroside, 3-methoxy-4-hydroxybenzoic acid	[45,46,47,48,49,50]
*P. kerneri* Dalla Torre (a.n.)	aerial parts	verbascoside, leucosceptoside A, echinacoside, aucubin, monomelittoside, plantarenaloside, euphroside, mussaenosidic acid, 8-*epi*-loganic acid, D-mannitol	[51]
*P. langsdorffii* Fisch. ex. Steven (a.n.)	leaves	alkaloids, tannins (**exact compounds not specified**)	[24]
*P. lapponica* L. (a.n.)	aerial parts	alkaloids (**exact compounds not specified**), euphroside, aucubin, mussaenoside	[24,52]
*P. lasiophrys* Maxim. (a.n.)	whole plant	verbascoside, leucosceptoside A, cistanoside D, pedicularioside E, pedicularioside F, 8-*epi*-loganin	[53]
*P. longiflora* Rudolph (a.n.)	whole plant	scopoletin, longifloroside A, longifloroside B, longifloroside C, longifloroside D, 7(*R*)-dehydro-diconiferyl alcohol-4-*O*-β-d-glucoside, longiflor A, longiflor B, tortoside D, tortoside E, verbascoside, *iso*-verbascoside, leucosceptoside A, pedicularioside A, pedicularoside I, pedicularoside M, cistanoside D, echinacoside, geniposidic acid, mussaenoside, loganic acid, longifloroside, adenosine, 6-(1′′,3′′-dihydroxy-2′′-propoxyl)-inosine	[47,54,55,56]
*P. longiflora* var. *tubiformis* (Klotzsch) Tsoong (a.n.)	whole plant	hexatriacontanol, nonatriacontanol, 1-hydroxy-xanthone, β-daucosterol, martynoside, apigenin, chrysoeriol, luteolin, tricin, acacetin, orientin, morelosin, apigenin-7-*O*-glucuronide, luteolin-7-*O*-glucoside, luteolin-5-*O*-glucoside, chrysoeriol-7-*O*-glucuronide, luteolin-7-*O*-glucuronide, tricin-7-*O*-glucuronide, 7-deoxy-8-*epi*-loganic acid, mussaenosidic acid, boschnaloside, aucubin, muconic acid, cinnamic acid, *p*-formyl cinnamic acid	[57,58,59]
*P. muscicola* Maxim. (a.n.)	whole plant	hentriacontane, arachidic acid, β-daucosterol, syringaresinol-4-*O*-β-d-glucoside, verbascoside, martynoside, *cis*-martynoside, pedicularioside A, mussaenoside, euphroside, geniposidic acid, aucubin, mussaenosidic acid, shanzhiside methyl ester, penstemonoside, pedicularioside, gardoside methyl ester, sesamoside, phloyoside II, caryoptoside, d-mannitol	[60,61,62]
*P. nordmanniana* Bunge (u.n.)	aerial parts	verbascoside, martynoside, leucosceptoside A, forsythoside B, iridolactone, geniposidic acid, aucubin, euphroside, mussaenoside	[63]
*P. palustris* L.	aerial parts	aucubin, euphroside, ixoroside, shanzhiside methyl ester, gardoside methyl ester, plantarenaloside, mussaenoside, pedicularioside, penstemonoside, boschnaloside, 8-*epi*-loganin, 7-deoxy-8-*epi*-loganin, 8-*epi*-loganic acid	[52]
*P. pectinata* Wall. ex. Benn. (a.n.)	flowers	phenolics (**exact compounds not specified**)	[21]
*P. peduncularis* Popov (a.n.)	aerial parts	plantagonine, indicainine, plantagonin, indicine, peducularine, *N*-methyl-cytisine	[64]
*P. plicata* Maxim.(a.n.)	whole plant	verbascoside, martynoside, *iso*-martynoside, *cis*-leucosceptoside A, 3,4-dihydroxy-phenethyl alcohol, 1-*O*-β-d-(3,4-dihydroxy-β-phenylethyl)-glucopyranoside, boschnaloside, plicatoside A, plicatoside B	[65]
*P. procera* A. Gray (u.n.)	aerial parts	aucubin, mussaenoside, 6-deoxy-catalpol, shanzhiside methyl ester, 8-*epi*-loganic acid, gardoside, proceroside	[22,66]
*P. punctata* Decne. (a.n.)	flowers, leaves	phenolics (**exact compounds not specified**), verbascoside, aucubin	[8,67]
*P. pycnantha* Boiss. (u.n.)	whole plant	alkaloids, tannins (**exact compounds not specified**)	[68]
*P. racemosa* Douglas ex. Benth. (a.n.)	aerial parts	lupanine, tetrahydrorhombifoline, aucubin, euphroside	[22,30]
*P. resupinata* L. (a.n.)	whole plant	alaschanioside A, alaschanioside C, syringaresinol-4′′-*O*-β-d-glucoside, verbascoside, 2′′′,3′′′-*O*-diacetyl-martynoside, leucosceptoside A, plantarenaloside, euphroside, boschnaloside, gardoside methyl ester, geniposidic acid	[16,49]
*P. rex* C.B. Clarke ex. Maxim. (a.n.)	whole plant	verbascoside, martynoside, *iso*-martynoside, 4-hydroxy-phenylpropenyl-α-l-rhamnopyranosyl-(1→3)-4-*O*-feruloyl-β-d-glucopyranoside, apigenin, chrysoeriol, luteolin, luteolin-7-*O*-glucoside, 5,4′-di-hydroxy-3′-methoxy-flavone-7-*O*-6′′-*n*-butyryl-β-d-glucopyranoside, aucubin, 6-*O*-ethyl-aucubin, euphroside, 6-*O*-ethyl-*epi*-aucubin, mussaenoside, plantarenaloside, pedicurexoside	[69]
*P. rostratocapitata* Crantz (a.n.)	aerial parts	verbascoside, echinacoside, campneoside II, cistantubuloside C_1_, aucubin, euphroside, monomelittoside, mussaenosidic acid, 8-*epi*-loganic acid	[70]
*P. sarawschanica* Regel (u.n.)	fruits	plantagonine, peducularine	[71]
*P. semibarbata* A. Gray (a.n.)	whole plant	α-*iso*-lupanine, **17-oxo-*iso*-lupanine or isomer**	[72]
*P. semitorta* Maxim. (a.n.)	whole plant	syringaresinol-4′′-*O*-β-d-glucoside, semitortoside A, semitortoside B, *cis*-*iso*-verbascoside, shanzhiside methyl ester, mussaenoside	[73]
*P. sibthorpii*	aerial parts	verbascoside, martynoside, *iso*-martynoside, luteolin 7-*O*-glucoside, aucubin, d-mannitol	[74]
*P. siphonantha* D.Don (a.n.)	whole plant	(+)-dehydro-vomifoliol, vomifoliol, ω-hydroxy-propioguaiacone, 3-hydroxy-1-(4-hydroxy-3,5-dimethoxyphenyl)-1-propanone	[25]
*P. spicata* Pall. (a.n.)	whole plant	verbascoside, permethyl-verbascoside, pedicularioside A, pedicularioside G, pedicularioside H, *cis*-pedicularioside H, shanzhiside methyl ester, gardoside methyl ester, 5-deoxy-puchelloside I	[75,76]
*P. striata* Pall. (a.n.)	whole plant	ecdysterone 3-*O*-β-d-galactoside, striatoside A, striatoside B, verbascoside, *iso*-verbascoside, echinacoside, pedicularioside A, pedicularioside G, pedicularioside H, decaffeoyl-verbascoside, 1′-*O*-β-d-(3-methoxy-4-hydroxy-phenyl)-ethyl-α-l-apiosyl-(1→3′)-α-l-rhamnosyl-(1→6′)-4′-*cis*-feruloyl-glucopyranoside, 8-*O*-acetyl-harpagide, dihydro-catalpolgenin	[77,78,79]
*P. striata* subsp. *aracnoidea* (Franch.) Tsoong	whole plant	verbascoside, pedicularioside A, pedicularioside M, pedicularioside N, dihydro-catalpolgenin, eremophila-10,11-dien-7a,13-diol	[80,81,82]
*P. sudetica* Willd. (a.n.)	leaves	alkaloids (**exact compounds not specified**)	[24]
*P. sylvatica* L.(a.n.)	aerial parts	luteolin-7-*O*-glucoside, euphroside, plantarenaloside, 8-*epi*-loganin	[11,52]
*P. tenuirostris* Benth. (a.n.)	flowers and leaves	phenolics (**exact compounds not specified**)	[8]
*P. torta* Maxim. (a.n.)	whole plant	longiflor A, longiflor B, tortoside A, tortoside B, tortoside C, tortoside D, tortoside E, tortoside F, dihydro-dehydro-diconiferyl alcohol-4-*O*-α-l-rhamnoside, dihydro-dehydro-diconiferyl alcohol-4-*O*-β-d-glucoside, dihydro-dehydro-diconiferyl alcohol-9-*O*-β-d-glucoside, (7*R*)-dehydro-diconiferyl alcohol-4-*O*-β-d-glucoside, (7*S*)-dehydro-diconiferyl alcohol-4-*O*-β-d-glucoside, verbascoside, leucosceptoside A, cistanoside D, shanzhiside methyl ester, gardoside methyl ester, 8-*epi*-loganin, loganic acid	[83,84]
*P. tricolor* Hand.-Mazz. (a.n.)	whole plant	pedicutricone A, 3β,19α-dihydroxy-12-ursen-28-oic acid, β-sitosterol, β-daucosterol, verbascoside, martynoside, quercetin-7-*O*-galactoside, apigenin, luteolin, chrysoeriol, 3,3′-di-*O*-methyl-quercetin, 3,5,4′-trihydroxy-3′,5′-dimethoxy-flavone-7-*O*-β-d-glucopyranoside, 3,5,4′,5′-tetrahydroxy-3′-methoxy-flavone-7-*O*-β-d-glucopyranoside, 3,5,3′,4′-tetrahydroxy-flavone-7-*O*-β-glucopyranoside, myricetin-3′-methyl ester 7-*O*-glucopyranoside, pedicutricoside A, viburtinal, 3-methoxy-4-hydroxybenzoic acid	[85]
*P. uliginosa* Bunge (a.n.)	whole plant	(*rel*-4a*S*,7*R*,7a*R*)-1,4a,5,6,7,7a-hexahydro-7-hydroxyl-7-methyl-cyclopenta[c]pyran-4-carboxaldehyde, 1,3,5,6-tetrahydro-1-methoxyl-7-methyl-cyclopenta[c]pyran-4-carboxaldehyde, (*rel*-1*R*,4*S*,4a*S*,7*R*,7a*R*)-7-methyl-hexahydro-1,4-(epoxymethano)-cyclopenta[c]pyran-3(1*H*)-one, 4-*epi*-alyxialactone, alyxialactone, artselaenin A, artselaenin B, boschnarol, (4*R*)-4-hydroxymethyl- boschnialactone, densispicnin B	[86]
*P. verticillata* L. (a.n.)	whole plant	verticillatoside A, verticillatoside B, verbascoside, leucosceptoside A, cistanoside D, echinacoside, angoroside A, cistantubuloside B_1_, wiedemannioside C, excelside B, aucubin, euphroside, monomelittoside, mussaenosidic acid, 7-deoxy-8-*epi*-loganic acid, 8-*epi*-loganic acid, plantarenaloside, geniposidic acid, boschnaloside, caryoptoside, pediverticilatasin A, pediverticilatasin B, pediverticilatasin C, kansuenin B, densispicnin B, euphrasin, scyphiphin A1, scyphiphin A2, ligustroside	[70,87,88]
*P. wilhelmsiana*	aerial parts	phenolics (**exact compounds not specified**)	[12]

**Table 2:** Distribution of the Phytochemicals in the Various *Pedicularis* Species.

Phytochemical Class	Phytochemical Compound	*Pedicularis* spp.	References
Alkanes	hentriacontane	*P. muscicola*	[62]
Alkyl alcohols	hexatriacontanol	*P. longiflora* var. *tubiformis*	[58,59]
	nonatriacontanol	*P. longiflora* var. *tubiformis*	[58,59]
Fatty acids	arachidic acid	*P. muscicola*	[62]
Coumarins	scopoletin	*P. longiflora*	[47,56]
Xanthones	1-hydroxy-xanthone	*P. longiflora* var. *tubiformis*	[58,59]
	pedicutricone A	*P. densispica* *P. tricolor*	[38,85]
Terpenoids	1,2,3,16,19,20-hexahydroxyolean-12-en-28-oic acid	*P. kansuensis*	[50]
	3β,19α-dihydroxy-12-ursen-28-oic acid	*P. tricolor*	[85]
	β-sitosterol	*P. decora* *P. kansuensis* *P. tricolor*	[32,33,34,35,36,45,85]
	β-daucosterol	*P. decora* *P. kansuensis* *P. longiflora* var. *tubiformis* *P. muscicola* *P. tricolor*	[32,33,34,35,36,45,58,59,62,85]
	ecdysterone 3-*O-*β-d-galactoside	*P. striata*	[78]
	kidjolanin	*P. cephalantha*	[25]
Alkaloids	α-*iso*-lupanine	*P. semibarbata*	[72]
	anagyrine	*P. crenulata*	[30,42]
	indicaine	*P. dolichorrhiza* *P. dolichocymba*	[30,42]
	indicainine	*P. peduncularis*	[64]
	indicine	*P. peduncularis*	[64]
	lupanine	*P. racemosa*	[30]
	*N*-methyl-cytisine	*P. grayi* *P. peduncularis*	[30,64]
	peducularine	*P. peduncularis* *P. sarawschanica*	[64,71]
	pediculidine	*P. dolichocymba* *P. dolichorrhiza*	[30,42]
	pediculine	*P. dolichocymba* *P. dolichorrhiza*	[30,42]
	plantagonin	*P. peduncularis*	[64]
	plantagonine	*P. dolichocymba* *P. dolichorrhiza* *P. peduncularis* *P. sarawschanica*	[30,42,64,71]
	senecionine	*P. groenlandica*	[30]
	tetrahydrorhombifoline	*P. racemosa*	[30]
Lignans and *neo*-lignans	7(*R*)-dehydro-diconiferyl alcohol-4-*O*-β-d-glucoside	*P. longiflora* *P. torta*	[54,83]
	(7*S*)-dehydro-diconiferyl alcohol-4-*O*-β-d-glucoside	*P. torta*	[84]
	(+)-isolariciresinol 3a-*O*-β-d-glucopyranoside	*P. densispica*	[39]
	alaschanioside A	*P. alaschanica* *P. artselaeri* *P. kansuensis* *P. resupinata*	[17,19,47,49,50]
	alaschanioside C	*P. alaschanica* *P. kansuensis* *P. resupinata*	[17,47,49,50]
	armaoside	*P. armata*	[18]
	citrusin A	*P. alaschanica* *P. artselaeri*	[17,19]
	citrusin B	*P. armata*	[17,18]
	densispicoside	*P. densispica*	[39]
	dihydro-dehydro-diconiferyl alcohol-4-*O*-β-d-glucoside	*P. torta*	[84]
	dihydro-dehydro-diconiferyl alcohol-4-*O*-α-l-rhamnoside	*P. torta*	[84]
	dihydro-dehydro-diconiferyl alcohol-9-*O*-β-d-glucoside	*P. torta*	[84]
	lariciresinol-4-*O*-β-d-glucoside	*P. artselaeri*	[19]
	lariciresinol-4′-*O*-β-d-glucoside	*P. artselaeri* *P. dolichocymba*	[19,41]
	longiflor A	*P. longiflora* *P. torta*	[54,83]
	longiflor B	*P. longiflora* *P. torta*	[54,83]
	longifloroside A	*P. longiflora*	[54]
	longifloroside B	*P. densispica* *P. longiflora*	[39,54]
	longifloroside C	*P. longiflora*	[54]
	longifloroside D	*P. longiflora*	[54]
	pinoresinol	*P. cephalantha*	[25]
	semitortoside A	*P. semitorta*	[73]
	semitortoside B	*P. semitorta*	[73]
	pinoresinol-4-*O*-β-d-glucoside	*P. densispica*	[39]
	striatoside A	*P. striata*	[78]
	striatoside B	*P. striata*	[78]
	syringaresinol-4-*O*-β-d-glucoside	*P. alaschanica* *P. chinensis* *P. densispica* *P. muscicola*	[17,28,39,60,61]
	syringaresinol-4′′-*O*-β-d-glucoside	*P. resupinata* *P. semitorta*	[49,73]
	tortoside A	*P. torta*	[84]
	tortoside B	*P. torta*	[84]
	tortoside C	*P. torta*	[84]
	tortoside D	*P. longiflora* *P. torta*	[54,84]
	tortoside E	*P. longiflora* *P. torta*	[54,84]
	tortoside F	*P. torta*	[84]
	verticillatoside A	*P. verticillata*	[88]
	verticillatoside B	*P. verticillata*	[88]
Phenylethanoid glycosides	1-(2,3,4-trihydroxyphenyl)ethyl-3-*O*-rhamnose-4-[(2*E*)-3-(3,4-dihydroxyphenyl)-2-propenoate]-glucopyranoside	*P. kansuensis*	[47,49,50]
	1-(2,3,4-trihydroxyphenyl)ethyl-3-*O*-rhamnose-4-[(2*E*)-3-(3,4-dihydroxyphenyl)-2-propenoate]-6-[(2*E*)-3-(3,4-dihydroxyphenyl)-2-propenoate]-glucopyranoside	*P. kansuensis*	[47,49,50]
	1-*O*-β-d-(3,4-dihydroxy-β-phenylethyl)-glucopyranoside	*P. plicata*	[65]
	1-*O*-β-d-(3-hydroxy-4-methoxy-phenyl)-ethyl-β-d-apiosyl-l-(1→3)-rhamnosyl-(1→6)-4-*trans*-feruloyl-glucopyranoside	*P. chinensis*	[28]
	1-*O*-β-d-(3-hydroxy-4-methoxy-phenyl)-ethyl-β-1-(1→3)-4-*trans*-feruloyl-glucopyranoside	*P. chinensis*	[28]
	1-*O*-β-d-(3-hydroxy-4-methoxy-phenyl)-ethyl-α-l-rhamnosyl(1→3)-4-*cis*-feruloyl-gulopyranoside	*P. chinensis*	[28]
	1′-*O*-β-d-(3-methoxy-4-hydroxy-phenyl)-ethyl-α-l-apiosyl-(1→3′)-α-l-rhamnosyl-(1→6′)-4′-*cis*-feruloyl-glucopyranoside	*P. striata*	[78]
	2-(*p*-hydroxyphenyl)-ethanol-1-*O*-β-d-glucopyranoside	*P. artselaeri*	[19]
	2-phenylethyl-*O*-β-d-xylopyranosyl-(1→2)-β-d-glucopyranoside	*P. dolichocymba*	[41]
	2′′-*O*-acetyl-verbascoside	*P. densispica*	[39]
	2′′′-*O*-acetyl-martynoside	*P. dolichocymba*	[41]
	2′′′,3′′′-*O*-diacetyl-martynoside	*P. kansuensis* *P. resupinata*	[47,49,50]
	3,4-dihydroxy-phenethyl alcohol	*P. plicata*	[65]
	3-(4-hydroxy-3-methoxyphenyl)-1,2,3-propantriol	*P. densispica*	[39]
	4-hydroxy-phenylpropenyl-α-l-rhamnopyranosyl-(1→3)-4-*O*-feruloyl-β-d-glucopyranoside	*P. rex*	[69]
	4-*O*-β-d-glucopyranosyl-sinapic acid methyl ester	*P. densispica*	[39]
	angoroside A	*P. verticillata*	[70]
	artselaeroside A	*P. artselaeri*	[20]
	artselaeroside B	*P. artselaeri*	[20]
	benzyl alcohol-*O*-β-d-xylopyranosyl-(1→2)-β-d-glucopyranoside	*P. dolichocymba*	[41]
	*cis*-*iso*-martynoside	*P. kansuensis*	[47,49,50]
	*cis*-*iso*-verbascoside	*P. semitorta*	[73]
	*cis*-leucosceptoside A	*P. plicata*	[65]
	*cis*-martynoside	*P. chinensis* *P. densispica* *P. muscicola*	[28,39,62]
	*cis*-pedicularioside H	*P. spicata*	[76]
	campneoside II	*P. rostratocapitata*	[70]
	cistanoside D	*P. lasiophrys* *P. longiflora* *P. torta* *P. verticillata*	[53,55,84,88]
	cistantubuloside B_1_	*P. verticillata*	[70]
	cistantubuloside C_1_	*P. rostratocapitata*	[70]
	citrusin C	*P. densispica*	[39]
	clerodenoside A	*P. cephalantha*	[25]
	darendoside B	*P. densispica*	[39]
	decaffeoyl-verbascoside	*P. striata*	[77]
	forsythoside B	*P. comosa* *P. kansuensis* *P. nordmanniana*	[15,47,49,50,63]
	echinacoside	*P. acmodonta* *P. condensata* *P. kansuensis* *P. kerneri* *P. longiflora* *P. rostratocapitata* *P. striata* *P. verticillata*	[15,29,47,49,50,51,56,70,77]
	excelside B	*P. verticillata*	[70]
	*iso*-martynoside	*P. cephalantha* *P. densispica* *P. kansuensis* *P. plicata* *P. rex* *P. sibthorpii*	[25,47,49,50,65,69,74]
	*iso*-verbascoside	*P. artselaeri* *P. decora* *P. longiflora* *P. striata*	[20,33,47,56,77]
	jionoside B1	*P. kansuensis*	[47,49,50]
	jionoside D	*P. dolichocymba*	[41]
	leucosceptoside A	*P. acmodonta* *P. alaschanica* *P. dolichocymba* *P. kansuensis* *P. kerneri* *P. lasiophrys* *P. longiflora* *P. nordmanniana* *P. resupinata* *P. torta* *P. verticillata*	[15,17,41,45,47,49,50,51,53,55,63,84,88]
	martynoside	*P. alaschanica* *P. artselaeri* *P. cephalantha* *P. chinensis* *P. densispica* *P. dolichocymba* *P. kansuensis* *P. longiflora* var. *tubiformis* *P. muscicola* *P. nordmanniana* *P. plicata* *P. rex* *P. sibthorpii* *P. tricolor*	[17,20,25,28,39,41,45,47,49,50,57,62,63,65,69,74,85]
	pedicularioside A	*P. kansuensis* *P. longiflora* *P. muscicola* *P. spicata* *P. striata* *P. striata* subsp. *aracnoidea*	[47,49,50,56,62,75,77,82]
	pedicularioside E	*P. lasiophrys*	[53]
	pedicularioside G	*P. spicata* *P. striata*	[76,79]
	pedicularioside H	*P. spicata* *P. striata*	[75,78]
	pedicularioside I	*P. longiflora*	[55]
	pedicularioside M	*P. kansuensis* *P. longiflora* *P. striata* subsp. *aracnoidea*	[47,49,50,56,82]
	pedicularioside N	*P. chinensis* *P. striata* subsp. *aracnoidea*	[28,82]
	permethyl-verbascoside	*P. spicata*	[76]
	phenethylalcohol β-sophoroside	*P. kansuensis*	[45]
	robustaside B	*P. densispica*	[39]
	salidroside	*P. densispica*	[39]
	verbascoside	*P. alaschanica* *P. chamissonis* *P. comosa* *P. condensata* *P. densispica* *P. dolichocymba* *P. kansuensis* *P. kerneri* *P. lasiophrys* *P. longiflora* *P. muscicola* *P. nordmanniana* *P. plicata* *P. punctata* *P. resupinata* *P. rex* *P. rostratocapitata* *P. sibthorpii* *P. spicata* *P. striata* *P. striata* subsp. *aracnoidea* *P. tricolor* *P. torta* *P. verticillata*	[8,15,17,26,29,39,41,45,47,49,50,51,53,55,62,63,65,69,70,74,75,77,82,84,85,88]
	wiedemannioside C	*P. verticillata*	[70]
Flavonoids	3,3′-di-*O*-methyl-quercetin	*P. tricolor*	[85]
	3,5,4′-trihydroxy-3′,5′-dimethoxy-flavone-7-*O*-β-d-glucopyranoside	*P. tricolor*	[85]
	3,5,4′,5′-tetrahydroxy-3′-methoxy-flavone-7-*O*-β-d-glucopyranoside	*P. tricolor*	[85]
	3,5,3′,4′-tetrahydroxy-flavone-7-*O*-β-gluopyranoside	*P. tricolor*	[85]
	4′-methyl-chrysoeriol	*P. kansuensis*	[45]
	5,4′-di-hydroxy-3′-methoxy-flavone-7-*O*-6′′-*n*-butyryl-β-d-glucopyranoside	*P. rex*	[69]
	acacetin	*P. cephalantha* *P. densispica* *P. longiflora* var. *tubiformis*	[25,38,57]
	apigenin	*P. dolichocymba* *P. longiflora* var. *tubiformis* *P. rex* *P. tricolor*	[41,57,69,85]
	apigenin-7-*O*-glucoside	*P. densispica*	[38]
	apigenin-7-*O*-glucuronide	*P. longiflora* var. *tubiformis*	[57]
	chrysoeriol	*P. longiflora* var. *tubiformis* *P. rex* *P. tricolor*	[57,69,85]
	chrysoeriol-7-*O*-glucoside	*P. densispica*	[38]
	chrysoeriol-7-*O*-glucuronide	*P. longiflora* var. *tubiformis*	[57]
	kaempferol	*P. decora* *P. densispica*	[32,33,34,35,36,38]
	kaempferol-3,7-*O*-α-di-rhamnopyranoside	*P. densispica*	[38]
	lagotiside	*P. kansuensis*	[45]
	luteolin	*P. cephalantha* *P. kansuensis* *P. longiflora* var. *tubiformis* *P. rex* *P. tricolor*	[25,45,57,69,85]
	luteolin-5-*O*-glucoside	*P. longiflora* var. *tubiformis*	[57,58,59]
	luteolin-7-*O*-glucoside	*P. chamissonis* *P. chinensis* *P. kansuensis* *P. longiflora* var. *tubiformis* *P. rex* *P. sibthorpii* *P. sylvatica*	[11,26,28,45,57,69,74]
	luteolin-7-*O*-glucuronide	*P. chamissonis* *P. longiflora* var. *tubiformis*	[26,57]
	morelosin	*P. longiflora* var. *tubiformis*	[57,58,59]
	myricetin-3′-methyl ester 7-*O*-glucopyranoside	*P. tricolor*	[85]
	orientin	*P. longiflora* var. *tubiformis*	[57,58,59]
	quercetin-7-*O*-galactoside	*P. tricolor*	[85]
	scutellarein-7-*O*-glucoside	*P. densispica*	[38]
	tricin	*P. longiflora* var. *tubiformis*	[57]
	tricin-7-*O*-glucuronide	*P. kansuensis* *P. longiflora* var. *tubiformis*	[45,57]
Iridoids	1,3,5,6-tetrahydro-1-methoxyl-7-methyl-cyclopenta[c]pyran-4-carboxaldehyde	*P. uliginosa*	[86]
	3β-butoxy-3,4-dihydro-aucubin	*P. chinensis*	[27]
	4-*epi*-alyxialactone	*P. uliginosa*	[86]
	(*4R*)-4-hydroxymethyl-boschnialactone	*P. uliginosa*	[86]
	5-deoxy-puchelloside I	*P. spicata*	[75]
	6-*O*-acetyl-aucubin	*P. condensata*	[29]
	6-*O*-methyl-aucubin	*P. artselaeri* *P. chinensis*	[20,28]
	6-*O*-butyl-aucubin	*P. chinensis*	[27]
	6-*O*-butyl-*epi*-aucubin	*P. chinensis*	[27]
	6-*O*-ethyl-aucubin	*P. rex*	[69]
	6-*O*-ethyl-*epi*-aucubin	*P. rex*	[69]
	6-*O*-methyl-*epi*-aucubin	*P. artselaeri*	[20]
	6-deoxy-catalpol	*P. procera*	[22]
	7-*O*-acetyl-gardoside methyl ester	*P. dolichocymba*	[41]
	7-deoxy-8-*epi*-loganic acid	*P. artselaeri* *P. kansuensis* *P. palustris* *P. longiflora* var. *tubiformis* *P. verticillata*	[19,46,52,57,88]
	7-deoxy-gardoside	*P. artselaeri* *P. cephalantha*	[20,25]
	8-*O*-acetyl-harpagide	*P. striata*	[77]
	8-*epi*-loganic acid	*P. armata* *P. artselaeri* *P. kansuensis* *P. kerneri* *P. palustris* *P. procera* *P. rostratocapitata* *P. verticillata*	[18,19,22,46,51,52,70,88]
	8-*epi*-loganin	*P. artselaeri* *P. condensata* *P. densispica* *P. lasiophrys* *P. palustris* *P. sylvatica* *P. torta*	[20,29,39,52,53,84]
	(*rel*-1*R*,*4S*,4a*S*,7*R*,7a*R*)-7-methyl-hexahydro-1,4-(epoxymethano)-cyclopenta[c]pyran-3(1*H*)-one	*P. uliginosa*	[86]
	(*rel*-4a*S*,7*R*,7a*R*)-1,4a,5,6,7,7a-hexahydro-7-hydroxyl-7-methyl-cyclopenta[c]pyran-4-carboxaldehyde	*P. uliginosa*	[86]
	*rel*-(6*R*,5*R*,9*R*)-(2-oxa-bicyclo-[3,3,0]oct-3-one-8-en-9,8-diyl)-dimethanol	*P. chinensis*	[28]
	alyxialactone	*P. uliginosa*	[86]
	argyol	*P. densispica*	[37]
	artselaenin I	*P. artselaeri*	[19]
	artselaenin III	*P. artselaeri*	[19]
	artselaenin A	*P. artselaeri* *P. uliginosa*	[19,86]
	artselaenin B	*P. artselaeri* *P. uliginosa*	[20,86]
	artselaenin C	*P. artselaeri*	[20]
	aucubin	*P. armata* *P. artselaeri* *P. bracteosa* *P. cephalantha* *P. chinensis* *P. condensata* *P. crenulata* *P. decora* *P. groenlandica* *P. kansuensis* *P. kerneri* *P. lapponica* *P. longiflora* var. *tubiformis* *P. muscicola* *P. nordmanniana* *P. palustris* *P. procera* *P. punctata* *P. racemosa* *P. rex* *P. rostratocapitata* *P. sibthorpii* *P. verticillata*	[18,20,22,25,27,29,32,33,34,35,36,46,51,52,57,62,63,67,69,70,74,88]
	bartsioside	*P. chinensis*	[27]
	boschnaloside	*P. alaschanica* *P. longiflora* var. *tubiformis* *P. kansuensis* *P. palustris* *P. plicata* *P. resupinata* *P. verticillata*	[16,17,46,52,57,65,88]
	boschnarol	*P. uliginosa*	[86]
	caryoptoside	*P. artselaeri* *P. muscicola* *P. verticillata*	[20,60,61,88]
	densispicnin A	*P. densispica*	[37]
	densispicnin B	*P. densispica* *P. uliginosa* *P. verticillata*	[37,86,87]
	densispicnin C	*P. densispica*	[39]
	densispicnin D	*P. densispica*	[39]
	dihydro-catalpolgenin	*P. striata* *P. striata* subsp. *aracnoidea*	[78,81]
	dolichocymboside A	*P. dolichocymba*	[40]
	dolichocymboside B	*P. dolichocymba*	[40]
	dolichocymboside C	*P. dolichocymba*	[40]
	dolichocymboside D	*P. dolichocymba*	[40]
	euphrasin	*P. verticillata*	[87]
	euphroside	*P. alaschanica* *P. armata* *P. cephalantha* *P. crenulata* *P. groenlandica* *P. kansuensis* *P. kerneri* *P. lapponica* *P. muscicola* *P. nordmanniana* *P. palustris* *P. racemosa* *P. resupinata* *P. rex* *P. rostratocapitata* *P. sylvatica* *P. verticillata*	[16,17,18,22,25,46,51,52,62,63,69,70,88]
	gardoside	*P. procera*	[22]
	gardoside methyl ester	*P. artselaeri* *P. condensata* *P. dolichocymba* *P. kansuensis* *P. muscicola* *P. palustris* *P. resupinata* *P. spicata* *P. torta*	[16,20,29,41,52,62,75,84]
	geniposidic acid	*P. alaschanica* *P. armata* *P. kansuensis* *P. longiflora* *P. muscicola* *P. nordmanniana* *P. resupinata* *P. verticillata*	[16,17,18,46,48,55,62,63,88]
	iridolactone	*P. chinensis* *P. nordmanniana*	[27,63]
	ixoroside	*P. alaschanica* *P. artselaeri* *P. kansuensis* *P. palustris*	[16,17,20,46,52]
	kansuenin	*P. kansuensis*	[46]
	kansuenin B	*P. kansuensis* *P. verticillata*	[45,87]
	kansuenoside	*P. kansuensis*	[46]
	lamalbid	*P. decora*	[32,33,34,35,36]
	ligustroside	*P. verticillata*	[70]
	loganic acid	*P. longiflora* *P. torta*	[55,84]
	longifloroside	*P. longiflora*	[55]
	monomelittoside	*P. kerneri* *P. rostratocapitata* *P. verticillata*	[51,70]
	mussaenin A	*P. densispica*	[37]
	mussaenoside	*P. armata* *P. artselaeri* *P. bracteosa* *P. cephalantha* *P. condensata* *P. densispica* *P. groenlandica* *P. kansuensis* *P. lapponica* *P. longiflora* *P. muscicola* *P. nordmanniana* *P. palustris* *P. procera* *P. rex* *P. semitorta*	[18,19,22,25,29,37,46,52,55,62,63,69,73]
	mussaenosidic acid	*P. alaschanica* *P. cephalantha* *P. kerneri* *P. longiflora* var. *tubiformis* *P. muscicola* *P. rostratocapitata* *P. verticillata*	[16,17,25,51,57,62,70]
	ningpogoside B	*P. decora*	[32,33,34,35,36]
	pedicularioside	*P. muscicola* *P. palustris*	[52,62]
	pedicularioside F	*P. lasiophrys*	[53]
	pedicularislactone	*P. chinensis*	[27]
	pedicularislactone glucoside	*P. chinensis* *P. decora*	[28,32,33,34,35,36]
	pedicutricoside A	*P. tricolor*	[85]
	pediverticilatasin A	*P. verticillata*	[87]
	pediverticilatasin B	*P. verticillata*	[87]
	pediverticilatasin C	*P. verticillata*	[87]
	penstemonoside	*P. muscicola* *P. palustris*	[52,62]
	plantarenaloside	*P. artselaeri* *P. cephalantha* *P. crenulata* *P. decora* *P. kerneri* *P. palustris* *P. resupinata* *P. rex* *P. rostratocapitata* *P. sylvatica* *P. verticillata*	[16,19,22,25,37,51,52,69,88]
	phloyoside II	*P. muscicola*	[60,61]
	plicatoside A	*P. plicata*	[65]
	plicatoside B	*P. plicata*	[65]
	proceroside	*P. procera*	[66]
	scyphiphin A1	*P. verticillata*	[87]
	scyphiphin A2	*P. verticillata*	[87]
	sesamoside	*P. muscicola*	[60,61]
	shanzhiside methyl ester	*P. artselaeri* *P. condensata* *P. densispica* *P. muscicola* *P. palustris* *P. procera* *P. semitorta* *P. spicata* *P. torta*	[20,22,29,39,52,62,73,75,84]
	viburtinal	*P. tricolor*	[85]
Other	(+)-dehydro-vomifoliol	*P. siphonantha*	[25]
	2,5-dihydroxybenzoic acid	*P. decora*	[31]
	3-hydroxy-1-(4-hydroxy-3,5-dimethoxyphenyl)-1-propanone	*P. siphonantha*	[25]
	3-hydroxy-4-methoxybenzoic acid	*P. decora*	[31]
	3-methoxy-4-hydroxybenzoic acid	*P. decora* *P. kansuensis* *P. tricolor*	[31,45,47,49,50,85]
	3-methoxy-4-primeverosyl-acetophenone	*P. artselaeri*	[19]
	6-(1′′,3′′-dihydroxy-2′′-propoxyl)-inosine	*P. longiflora*	[47,56]
	β-(3′,4′-dihydroxyphenyl-*O*-a-l-rhamnopyranosyl-(1–3)-β-d-glucopyranoside	*P. decora*	[31]
	(*E*)-2-hexenyl β-sophoroside	*P. kansuensis*	[45]
	*p*-formyl cinnamic acid	*P. longiflora* var. *tubiformis*	[58,59]
	ω-hydroxy-propioguaiacone	*P. siphonantha*	[25]
	adenosine	*P. dolichocymba* *P. longiflora*	[41,47,56]
	alanine	*P. decora*	[31]
	arginine	*P. decora*	[31]
	aspartic acid	*P. decora*	[31]
	cinnamic acid	*P. longiflora* var. *tubiformis*	[58,59]
	cysteine	*P. decora*	[31]
	d-mannitol	*P. decora* *P. kerneri* *P. muscicola* *P. sibthorpii*	[31,51,62,74]
	dearabinosyl-pneumonanthoside	*P. densispica*	[38]
	eremophila-10,11-dien-7a,13-diol	*P. striata* subsp. *aracnoidea*	[80,81]
	glutamic acid	*P. decora*	[31]
	glycine	*P. decora*	[31]
	isoleucine	*P. decora*	[31]
	leucine	*P. decora*	[31]
	maltol-β-d-glucoside	*P. densispica*	[38]
	methionine	*P. decora*	[31]
	muconic acid	*P. longiflora* var. *tubiformis*	[58,59]
	pedicurexoside	*P. rex*	[69]
	phenylalanine	*P. decora*	[31]
	proline	*P. decora*	[31]
	salicylic acid	*P. decora*	[31]
	serine	*P. decora*	[31]
	threonine	*P. decora*	[31]
	tyrosine	*P. decora*	[31]
	uridine	*P. dolichocymba*	[41]
	valine	*P. decora*	[31]
	vomifoliol	*P. siphonantha*	[25]

**Table 3:** Ethnopharmacological Uses of *Pedicularis* Species as Reported in Literature.

*Pedicularis* spp.	Ethnopharmacological Uses	Organ/Form	Area of the World	References
*P. artselaeri*	to treat diuresis, exhaustion, collapse, senility	aerial parts/ n.r.	Northwestern China	[119]
*P. bicornuta*	- to treat vaginal and seminal discharges - to treat burns, rheumatism, gout, general inflammation, acidity	- inflorescence/ paste - whole plant/ decoction	- Nepal (Central Himalaya) - China, India	- [120] - [8,121]
*P. bifida* (Buch.-Ham.) Pennell (u.n.)	- to treat stomachache - to relieve joint paints	roots/ liquid and powder	Nepal (Newar community of Pharping Village, Kathmandu District)	[122]
*P. capitata*	- to sedate and relax - to stop bleeding in minor injuries	whole plant/ infusion	Canada (Inuit people of Kugluktuk, Nunavut regions)	[123]
*P. cheilanthifolia* Schrenk (a.n.)	- to cure stomachache, vaginal discharge, leucorrhoea, menorrhagia	whole plant, wood/ ethanolic extract, powder	India/Kashmir (Ladakh region)	[124,125]
*P. chenocephala* Diels (a.n.)	- to relieve pain - to treat oedema, oliguria, asthma, malnutrition, pains induced by osteomyelitis	flowers/ decoction	China	[8]
*P. chinensis*	- to nourish yin - to invigorate kidney - to strengthen spleen and stomach	roots/ decoction	China	[8]
*P. comosa*	- to be used as food stuff	flowers/ nectar	Turkey	[126]
*P. cranolopha* Maxim. (a.n.)	- to clear away heat evil - to expel superficial evils - to treat fever, urinary tract infections, hepatitis, pneumonia, sore pain due to external injury	whole plant/ decoction	China	[8]
*P. davidii* Franch. (a.n.)	- to strengthen spleen and stomach - to nourish yin - to relieve pain - to treat inanition, kidney deficiency, osteopyrexia, fever, joint pain, anorexia	rhizomes/ decoction	China	[8]
*P. decora*	- to treat general debility, collapse, exhaustion, seminal emission, spontaneous sweating and senility - to invigorate the mind and the circulation of blood - to strengthen spleen and stomach	roots/ decoction	China	[8,127]
*P. decorissima* Diels (a.n.)	- to clear away heat evil - to expel superficial evils - to treat acute gastroenteritis and food poisoning	whole plant, flowers/ decoction	China	[8]
*P. dissecta* (Bonati) Pennell & H.L. Li (a.n.)	- to supplement qi - to nourish yin - to detoxificate - to relieve pain - to treat asthenia due to disease, yin deficiency, sore, joint pains	roots/ decoction	China	[8]
*P. dunniana* Bonati (a.n.)	- to nourish yin - to relieve pain - to treat inanition, kidney deficiency, osteopyrexia, fever, joint pains, anorexia	rhizomes/ decoction	China	[8]
*P. flagellaris* Benth. (u.n.)	- to treat excessive diuresis and wounds - to treat excessive diuresis, wounds, rheumatisms - to regulate menstruation	- aerial parts/ infusion, decoction - aerial parts/infusion, decoction	- Himalaya - Bhutan	- [128] - [128]
*P. flava* Pall. (a.n.)	- to treat general body pains, stomachaches - to be used as sedative	leaves/ decoction	Pakistan	[129]
*P. gracilis*	to treat stomachache	roots/ liquid	Nepal (Newar community of Pharping village, Kathmandu District; Western regions)	[122,130]
*P. gracilis* subsp. *gracilis* (s.n.)	to relieve joint pain	roots /powder	Nepal (Central Himalaya)	[122]
*P. henryi* Maxim. (a.n.)	- to nourish yin and qi - to strengthen tendons and bones with vital essence - to activate collaterals - to treat hemiplegia and arthralgia due to blood stagnation	roots/ decoction	China	[8]
*P. hoffmeisteri* Klotzsch (a.n.)	- to cure flatulence and stomach disorders in animals - to cure food poisoning	- whole plant/ n.r. - whole plant/ n.r.	- India (Uttaranchal State) - India (Western Himalaya)	[131,132]
*P. integrifolia*	- to treat dropsy, excessive diuresis, asthma, rheumatisms - to heal wounds and oedema - to nourish body	aerial parts/ ethanolic extract	Bhutan	[44]
*P. kansuensis*	- to treat collapse, exhaustion, senility, edema and boils - to relieve heat and toxicity - to treat edema, inflammation, urinaryobstructions	- aerial parts/ n.r. - flowers/ n.r.	- China - Tibet, China	- [50,119] - [8]
*P. lanata* Willd. ex Cham. & Schltdl. (a.n.)	to treat headache, migraine	n.r./n.r.	Canada (Aborigens of the Boreal forest)	[133]
*P. longicaulis* Franch. ex Maxim. (a.n.)	- to nourish yin and qi - to activate collaterals - to treat dizziness tinnitus, bones and muscles pain, deficiency heat	roots/decoction	China	[8]
*P. longiflora*	- to cure hepatic, pancreatic, kidney, urinary diseases, vaginal discharge, leucorrhoea, menorrhagia - to treat rheumatisms, excessive diuresis and coagulation, wounds, hypertension, dehydration - to treat edema, tinnitus, carbuncles wollen, hepatitis, spermatorrhea, urine with pus and blood, cholecystitis, dry mouth, carbuncle swollen - to treat vertigo, dry tongue, excessive seminal discharge, edema, liver and gall bladder problems	- whole plant, wood/ decoction, powder - aerial parts/ ethanolic extract - whole plant, flowers/ decoction - leaves, stems/ decoctions	- Himalaya (Ladakh region) - Bhutan - China - India	- [7,125] - [44] - [8] - [121]
*P. longiflora* var. *tubiformis*	- to treat cough, sore throats, hepatitis, lymphatic disorders, poisioning, seminal and vaginal discharges, dropsy, spermatorrhoea, tinnitus, carbuncle disorders associated with alcoholism	whole plant/ raw food	Nepal (Central Himalaya)	[57,134]
*P. megalantha* D.Don (a.n.)	- to soothe meat poisoning, intestinal disorders, acidity	aerial parts/ decoction	Bhutan, Tibet	[135]
*P. megalochila* H.L. Li	to treat dysentery, diarrhea, hepatitis, urinary tract infections	whole plant/ decoction	China	[8]
*P. muscicola* Maxim. (a.n.)	- to nourish qi - to treat consumptiondiseases, blood deficiency, hidrosis, hypotension	roots/ decoction	China	[8]
*P. oederi* Vahl (a.n.)	- to treat rheumatic arthritis, lithangiuria, scabies, micturition difficulties - to treat food poisoning, headache, backache, bodyache - to be used as sedative	- roots/ decoction - whole plant/ raw vegetable	- China - India (Trans Himalaya region)	- [8] - [121,136]
*P. oederi* var. *sinensis* (Maxim.) Hurus. (a.n.)	to treat urinary obstructions and edema in animals	flowers/ n.r.	Tibet, China	[137]
*P. oliveriana* Prain (a.n.)	- to reduce inflammation - to ease gastric pains or disorders - to treat poisoning, micturition difficulties - to cure food poisoning, stomach ulcer, duodenal ulcer, diarrhea, rheumatic joint pains, lithangiuria, abnormal leucorrhea, scabies	- inflorescence/ extract - flowers, whole plant/ decoctions	- Nepal (Central Himalaya) - China	- [138] - [8]
*P. pectinata*	- to increase urine flow - to cure swelling and stomach pains due to intestinal infections - to alleviate stomach pain, flatulence, intestinal infections, intestinal swelling, high blood pressure, backache, bodyache, fever - to increase urine flow - to cure haemoptysis, alopecia	- aerial parts/ powdered raw food in cold water - flowers/ powdered raw food in cold water - flowers/ decoction	- Kashmir - Western Himalaya (Lahaul-Spiti tribe) - India, Kashmir	- [139] - [140] - [141,142,143]
*P. pectinatiformis* Bonati (a.n.)	- to relieve pain - to relax	leaves/ infusion	Pakistan (Gilgit-Baltistan region)	[144]
*P. peduncularis*	- to treat uterine bleeding - to favour diuresis - to treat various skin diseases	- aerial parts/ decoction - flowers/ decoction - aerial parts/ bath	Tajikistan	[64]
*P. punctata*	- to treat fever, cancer and premature graying of hair - to improve digestion - to control blood pressure - to treat hypertension, fever, gastrointestinal disorders - to relax skeletal muscles	- inflorescence/ extract - aerial parts/powdered raw food in cold water - flowers/ powder in cold water	- Nepal (Central Himalaya) - Western Himalaya (Lahaul-Spiti tribe) - Pakistan	- [138] - [140,145] - [146,147]
*P. pyramidata* Royle ex Benth. (a.n.)	to treat fluid retention, headache, bone inflammations, serous fluids accumulation	whole plant/ raw food	Nepal (Central Himalaya), India	[138,143]
*P. resupinata*	- to treat malignant abscesses - to treat rheumatoid arthritis, rheumatic pains, joint pains, scabies, micturition difficulties - to cure lithangiuria abnormal leucorrhea, acute gastroenteritis, food poisoning	- aerial parts/n.r. - roots, stem/ powder, decoctions	- South Korea - China	- [148] - [8]
*P. rex*	- to invigorate qi and blood - to strengthen spleen - to treat yin deficiency, hectic fever, rheumatism, cirrhosis, ascites - to cure smallpox, measles, seasonal prevalent diseases	roots, whole plant/ decoctions	China	[8]
*P. rhinanthoides* Schrenk (a.n.)	- to treat cough, sore throat, hepatitis, lymphatic disorders, poisoning - to treat diabetes	whole plant/ raw food - whole plant/ decoction	- Nepal (Central Himalaya) - India	- [138] - [149]
*P. rudis* Maxim. (a.n.)	- to nourish yin - to relieve pain - to treat inanition, kidney deficiency, osteopyrexia, fever, joint pain, anorexia	rhizomes/ decoction	China	[8]
*P. scullyana* Prain ex Maxim. (u.n.)	to remove pimples	whole plant/ paste	Nepal (Western regions)	[130]
*P. siphonantha*	to treat cough, sore throat, hepatitis, lymphatic disorders, poisoning	whole plant/ raw food	Nepal (Central Himalaya)	[134,150]
*P. spicata*	- to nourish qi - to treat consumption diseases, blood deficiency, hidrosis, hypotension	roots/decoction	China	[8]
*P. striata*	to treat kidney-yang deficiency, edema, micturition difficulties	whole plant/ decoction	China	[8]
*P. tenuirostris*	to cure swelling and stomach pain due to intestinal infections	flowers/ powdered raw food in cold water	Western Himalaya (Lahaul-Spiti tribe)	[140]
*P. torta*	to treat inflammations and urinary obstructions in animals	flowers/ n.r.	Tibet, China	[137]
*P. verticillata*	- to nourish qi - to treat consumption diseases, blood deficiency, hidrosis, hypotension	roots/decoction	China	[8]

**Table 4:** Pharmacological Activities of *Pedicularis* Species as Reported in Literature.

*Pedicularis* spp.	Pharmacological Properties	Organs/Forms	Collection Area	References
*P. artselaeri*	- strong antioxidant - hepatoprotective	- aerial parts/ butanol and water extracts - water and ethanolic extracts	China	- [154] - [155]
*P. cadmea* Boiss. (u.n.)	weak antibacterial	aerial parts/ methanolic extract	Turkey	[156]
*P. condensata*	antibacterial, weak antioxidant, antifungal	aerial parts/ essential oil	Turkey	[10]
*P. davidii*	- strong antioxidant - hepatoprotective	- rhizomes/ butanol and water extracts - water and ethanolic extracts	China	- [154] - [155]
*P. decora*	antioxidant, antidiabetic, hepatoprotective, anti-inflammatory	roots/ethanolic, n-butanol and water extracts	China	[7,157,158]
*P. flava*	medium antimicrobial	whole plant/ ethanolic extract	Mongolia	[159]
*P. longiflora*	antidiabetic, antioxidant, radical scavenging	whole plant/ ethanolic extract	Himalaya (Ladakh region), China	[7,56]
*P. olympica* Boiss. (u.n.)	weak antimicrobial	aerial parts/ methanolic extract	Turkey	[155]
*P. mexicana* Zucc. ex Bunge (a.n.)	antioxidant, medium cytotoxic	whole plant/ methanolic extract	Mexico	[160]
*P. sibthorpii*	strong antioxidant, free-radical scavenging, antibacterial	aerial parts/ methanolic extract	Iran	[74]
*P. wilhelmsiana*	strong antioxidant, antibacterial	aerial parts/ methanolic extract	Iran	[12]

**Table 5:** Other Uses of *Pedicularis* Species As Reported In Literature.

*Pedicularis* spp.	Other Uses	Organs/Forms	Area of Employment	References
*P. atuntsiensis* Bonati (a.n.)	purely ornamental	-	China (Northwestern Yunnan)	[170]
*P. capitata*	to make an olive green dye	flower stalks	Canada (Inuit people of Kugluktuk, Nunavut regions)	[123]
*P. crenularis* H.L. Li (a.n.)	purely ornamental	-	China (Northwestern Yunnan)	[170]
*P. cyclorhyncha* H.L. Li (a.n.)	purely ornamental	-	China (Northwestern Yunnan)	[170]
*P. dichrocephala* Hand.-Mazz. (a.n.)	purely ornamental	-	China (Northwestern Yunnan)	[170]
*P. fastigiata* Franch. (a.n.)	purely ornamental	-	China (Northwestern Yunnan)	[170]
*P. filicula* Franch. ex. Maxim. (a.n.)	purely ornamental	-	China (Northwestern Yunnan)	[170]
*P. flava*	forage	-	Pakistan	[129]
*P. gracilicaulis* H.L. Li (a.n.)	purely ornamental	-	China (Northwestern Yunnan)	[170]
*P. groenlandica*	edible plant	whole plant/tea	Canada (Inuit people, Kangiqsualujjuaq community)	[171]
*P. habachanensis* Bonati (a.n.)	purely ornamental	-	China (Northwestern Yunnan)	[170]
*P. humilis* Bonati (a.n.)	purely ornamental	-	China (Northwestern Yunnan)	[170]
*P. kariensis* Bonati (a.n.)	purely ornamental	-	China (Northwestern Yunnan)	[170]
*P. labradorica* Wirsing (a.n.)	edible plant	roots	Canada (Inuit people, Nain community)	[170]
*P. lamioides* Hand.-Mazz. (a.n.)	purely ornamental	-	China (Northwestern Yunnan)	[169]
*P. lanpingensis* H.P. Yang (a.n.)	purely ornamental	-	China (Northwestern Yunnan)	[169]
*P. lecomtei* Bonati (a.n.)	purely ornamental	-	China (Northwestern Yunnan)	[169]
*P. macrorhyncha* H.L. Li (a.n.)	purely ornamental	-	China (Northwestern Yunnan)	[170]
*P. maxonii* Bonati (a.n.)	purely ornamental	-	China (Northwestern Yunnan)	[170]
*P. mayana* Hand.-Mazz. (a.n.)	purely ornamental	-	China (Northwestern Yunnan)	[170]
*P. meteororhyncha* H.L. Li (a.n.)	purely ornamental	-	China (Northwestern Yunnan)	[170]
*P. micrantha* H.L. Li (a.n.)	purely ornamental	-	China (Northwestern Yunnan)	[170]
*P. mussotii* Franch. (a.n.)	purely ornamental	-	China (Northwestern Yunnan)	[170]
*P. obscura* Bonati (a.n.)	purely ornamental	-	China (Northwestern Yunnan)	[170]
*P. oederi*	fodder	whole plant/raw food	Nepal (Central Himalaya)	[138]
*P. oligantha* Franch. ex. Maxim. (a.n.)	purely ornamental	-	China (Northwestern Yunnan)	[170]
*P. orthocoryne* H.L. Li (a.n.)	purely ornamental	-	China (Northwestern Yunnan)	[170]
*P. pinetorum* Hand.-Mazz. (a.n.)	purely ornamental	-	China (Northwestern Yunnan)	[170]
*P. praeruptorum* Bonati (a.n.)	purely ornamental	-	China (Northwestern Yunnan)	[170]
*P. pseudoversicolor* Hand.-Mazz. (a.n.)	purely ornamental	-	China (Northwestern Yunnan)	[170]
*P. remotiloba* Hand.-Mazz. (a.n.)	purely ornamental	-	China (Northwestern Yunnan)	[170]
*P. salicifolia* Bonati (a.n.)	purely ornamental	-	China (Northwestern Yunnan)	[170]
*P. schizocalyx* (Lange) Steininger (a.n.)	edible	flowers/raw plant	Spain (Cantabria region)	[172]
*P. sigmoidea* Franch. ex. Maxim. (a.n.)	purely ornamental	-	China (Northwestern Yunnan)	[170]
*P. sylvatica*	edible	flowers/raw plant	Spain (Galicia region)	[170]
*P. tomentosa* H.L. Li (a.n.)	purely ornamental	-	China (Northwestern Yunnan)	[170]
*P. tsaii* H.L. Li (a.n.)	purely ornamental	-	China (Northwestern Yunnan)	[170]
*P. umbelliformis* H.L. Li (a.n.)	purely ornamental	-	China (Northwestern Yunnan)	[170]
*P. weixiensis* H.P. Yang (a.n.)	purely ornamental	-	China (Northwestern Yunnan)	[170]
*P. yui* H.L. Li (a.n.)	purely ornamental	-	China (Northwestern Yunnan)	[170]
*P. zhongdianensis* H.P. Yang (a.n.)	purely ornamental	-	China (Northwestern Yunnan)	[170]

## Abbreviations

a.n.	accepted name
n.r.	none reported
n.s.	not specified
s.n.	synonym name
u.n.	unresolved name
